# Platelet-rich plasma in orthopedic therapy: a comparative systematic review of clinical and experimental data in equine and human musculoskeletal lesions

**DOI:** 10.1186/s12917-015-0403-z

**Published:** 2015-04-22

**Authors:** Patrícia M Brossi, Juliana J Moreira, Thaís SL Machado, Raquel YA Baccarin

**Affiliations:** Department of Internal Medicine, School of Veterinary Medicine and Animal Science, University of São Paulo, São Paulo, SP Brazil

**Keywords:** Platelet-rich plasma, Systematic review, Horse, Tendon, Ligament, Joint

## Abstract

**Background:**

This systematic review aimed to present and critically appraise the available information on the efficacy of platelet rich plasma (PRP) in equine and human orthopedic therapeutics and to verify the influence of study design and methodology on the assumption of PRP’s efficacy. We searched Medline, PubMed, Embase, Bireme and Google Scholar without restrictions until July 2013. Randomized trials, human cohort clinical studies or case series with a control group on the use of PRP in tendons, ligaments or articular lesions were included. Equine clinical studies on the same topics were included independently of their design. Experimental studies relevant to the clarification of PRP’s effects and mechanisms of action in tissues of interest, conducted in any animal species, were selected.

**Results:**

This review included 123 studies. PRP’s beneficial effects were observed in 46.7% of the clinical studies, while the absence of positive effects was observed in 43.3%. Among experimental studies, 73% yielded positive results, and 7.9% yielded negative results. The most frequent flaws in the clinical trials’ designs were the lack of a true placebo group, poor product characterization, insufficient blinding, small sampling, short follow-up periods, and adoption of poor outcome measures. The methods employed for PRP preparation and administration and the selected outcome measures varied greatly. Poor study design was a common feature of equine clinical trials. From studies in which PRP had beneficial effects, 67.8% had an overall high risk of bias. From the studies in which PRP failed to exhibit beneficial effects, 67.8% had an overall low risk of bias.

**Conclusions:**

Most experimental studies revealed positive effects of PRP. Although the majority of equine clinical studies yielded positive results, the human clinical trials’ results failed to corroborate these findings. In both species, beneficial results were more frequently observed in studies with a high risk of bias. The use of PRP in musculoskeletal lesions, although safe and promising, has still not shown strong evidence in clinical scenarios.

**Electronic supplementary material:**

The online version of this article (doi:10.1186/s12917-015-0403-z) contains supplementary material, which is available to authorized users.

## Background

Musculoskeletal lesions are a common consequence of physical overstrain, which negatively impacts quality of life and athletic performance. Specifically among both, equine and human athletes, treating persistent or slow healing injuries poses a challenge for clinicians. These lesions, which frequently result in inadequate tissue reorganization and thus in a high re-injury rate, are often related to a long period of incapacity or to an unsatisfactory return to performance [1].

Hemoderivatives have been reported to be beneficial in scenarios in which efficient, cost-effective and safe forms of orthopedic interventions are necessary to restore the normal function and structure of musculoskeletal components and in which health care professionals are tempted to explore promising forms of therapy. The use of patients’ own biological materials for tissue healing and therapeutic purposes offers a safe and interesting alternative to conventional treatments, and such materials often lack side effects. Presently, several blood-derived products are available for intra-lesional injection, such as platelet-rich plasma (PRP) or plasma rich in growth factors, autologous conditioned serum, autologous blood preparations and autologous protein concentrate [1-6].

The rationale behind the injection of autologous blood preparations lies in the exploitation of advantageous mechanisms of the body’s natural response to injury, whether the platelets’ ability to induce hemostasis and to release growth factors [7,8] or the production of anti-inflammatory cytokines by blood components [5,9]. Blood is an important and unique source of cellular and protein products that has been explored more intensively over the last three decades for the production of biomaterials for clinical use [4]. A considerable number of data have suggested beneficial effects associated with their use, but these findings have not been unanimous when experimental and clinical trial’ results have been compared.

Platelet-rich products, in particular, have gained popularity for their increased concentrations of growth factors, but their compositions encompass much more than these factors. A myriad of other blood-derived substances such as fibrin and leukocytes are contained within these products, characterizing PRP as a complex and unique mixture, with donor-related properties and a yet unrevealed spectrum of active blood components [10]. Platelet concentrates with distinct compositions, and therefore distinct applications, are referred to as PRP. Some authors have proposed a more accurate terminology, along with a thorough description of the platelet concentrate. Using this terminology four different products can be recognized according to their fibrin architecture and leukocyte content: pure platelet-rich plasma, leukocyte and platelet-rich plasma, pure platelet-rich fibrin and leukocyte- and platelet-rich fibrin [11].

The cellular and molecular content of platelet concentrates also depend on the method or kit used for their preparation. Until now, there has been no consensus on which processing method yields the best platelet- related product for a particular purpose, and patented and non-patented technologies and procedures have been used in clinical and experimental scenarios [12].

During athletics, both horses and humans are subjected to escalating stresses to their appendicular systems, to compete successfully. These increasing demands are accompanied by a multitude of orthopedic lesions, for which conventional therapies have proven partially helpful.

The objectives of this systematic review were to assess the effectiveness of PRP in the healing of tendon, ligament and articular lesions in equine and human patients, to compare the results from clinical and experimental studies in both species, and to verify the existence of a relationship between the study designs and results of clinical trials. This review also aimed to evaluate critically the existing evidence to provide clinicians with high-quality information and a more thorough appreciation of the effects of the diversity of PRP’ processing techniques.

## Methods

This systematic review was performed in accordance with the PRISMA (Preferred Reporting Items for Systematic Reviews and Meta-Analyses) statement, an evidence-based, established guideline for systematic reviews published by the CONSORT group [13,14].

A comprehensive literature search addressing the use of hemoderivatives in orthopedic lesions was conducted in July 2013 for all relevant articles in English, French, German, Spanish and Portuguese without publication or date restrictions, and all of the authors (PMB, JJM, TSLM, RYAB) were involved. The Embase, Bireme, Medline, PubMed and Google Scholar databases were used, with the search terms “platelet rich plasma”, “PRP”, “tendon”, “joint”, “articular”, “ligament”, “musculoskeletal injuries”, “human” and “equine”. Additional studies were identified by searching the reference lists of eligible articles. Studies that used PRP in conjunction with stem cells or other biomaterials, as regenerative/anti-inflammatory therapies for other target tissues (e.g., bone) or in other medical fields (such as ophthalmology, craniomaxillofacial or plastic surgery) were excluded.

Equine clinical studies, because of their scarcity, were included independently of their design or level of evidence if they described the effects of PRP on tendon, ligament or articular injuries. Human clinical studies were included if they reported the use of PRP in tendon, ligament or articular lesions and were either double-blind RCTs or prospective/retrospective cohort studies or if they were case series with a control group.

Experimental studies with PRP conducted in several species, both *in vivo* and *in vitro*, were selected if they were controlled and relevant to the clarification of the effects and mechanisms of action of PRP in tissues of interest. Studies that used products not derived from blood processing, despite an analogous function or a similar composition to PRP, were not included [15-17].

Preliminarily, the abstracts and titles were reviewed to select manuscripts for full-text review. Relevant data were then extracted from the selected articles based on predefined data fields and were sorted in tables corresponding to clinical and experimental studies, independently, by all of the authors. The tables were intended to (1) facilitate the identification of the lesion/tissue/population studied; (2) characterize the control and intervention groups; (3) state the outcome measures and methods employed for the evaluation of effects; (4) describe in detail the method of preparation, composition and protocol of administration of PRP; and (5) present the results and rate them as positive or beneficial (+), partially positive (±) or negative (impartial) (−). This assessment was not made by the review’s authors and is in accordance to the results presented, when confronted with the selected outcome measures. This overall view of the studies’ design and contents was followed by annotation of weak points that could have negatively affected the generation of quality evidence or that could have biased the studies’ results. Experimental studies with *in vivo* and *in vitro* ramifications had their results presented on separate lines to allow for a thorough analysis.

The risk of bias of the selected clinical trials was presented in tables according to PRISMA guidelines, and the studies were assigned as having a high or a low risk of bias if they exhibited, respectively, more or fewer bias criteria. This information, together with the analysis of the clinical trials’ results, was used to verify a possible association between these two variables and was also presented in tables.

## Results

**Figure 1:**
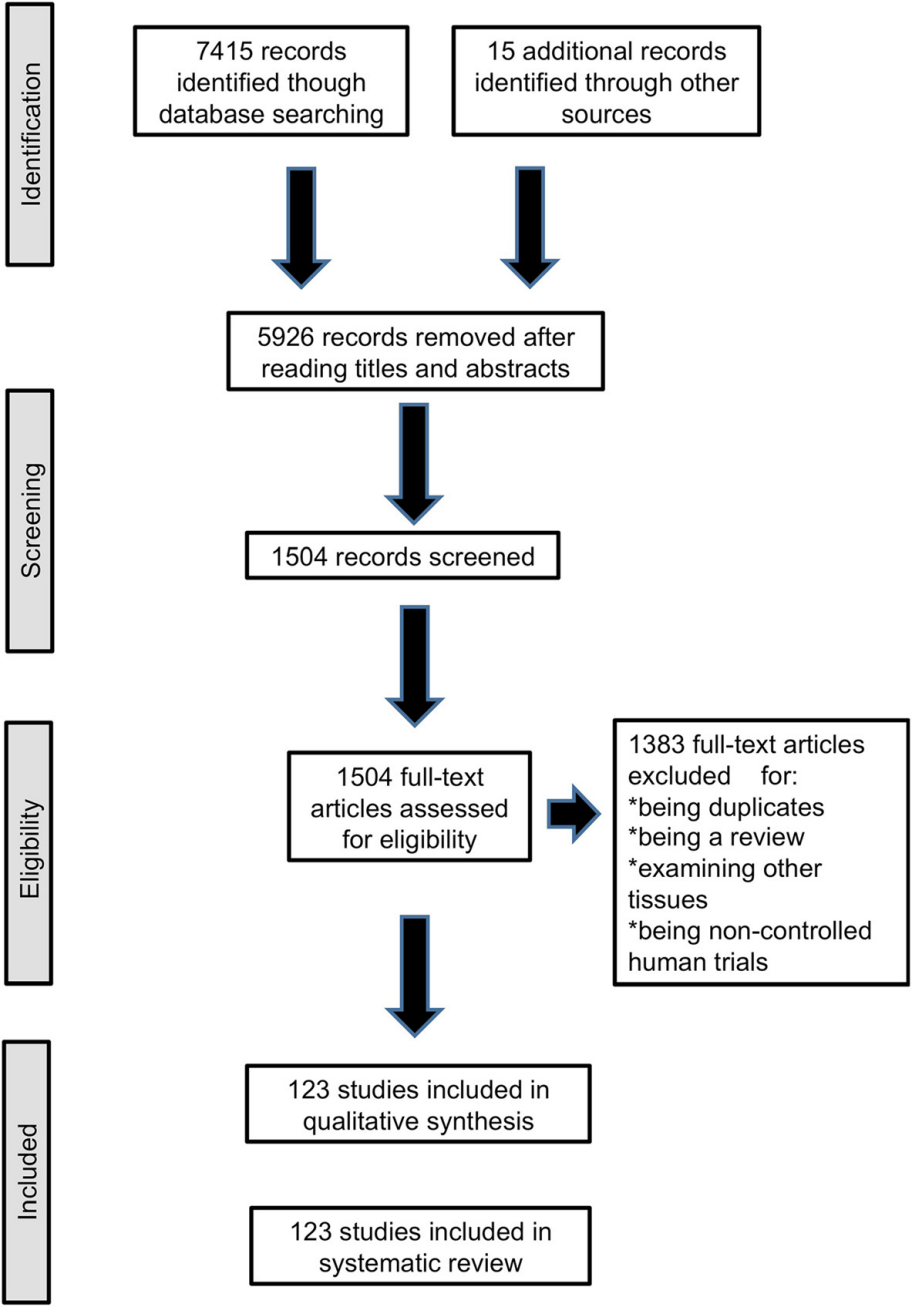
Flow diagram for identification of published studies. PRISMA 2009.

Our search parameters yielded 7415 results: 3563 from Embase, 2817 from Bireme, 101 from Medline, 122 from PubMed and 812 from Google Scholar. Fifteen studies were identified in reference lists from the selected articles. The titles and abstracts of the retrieved records were screened for eligibility, and 5926 studies were thus excluded. One thousand five hundred four articles were fully assessed. One hundred twenty-three (123) articles were selected after the exclusion criteria (i.e., the exclusion of duplicates, review articles, non-controlled human trials, studies in which other tissues were examined and experimental studies investigating PRP preparation particularities) were applied (Figure 1).

Among these 123 studies, a relatively homogeneous distribution between clinical trials (60 studies or 48.8%) and experimental studies (63 studies or 51.2%) was observed. These 123 articles yielded 126 results because two experimental studies comprised both *in vivo* and *in vitro* experiments, which were considered separately.

### Clinical trials with PRP

From the 60 clinical studies included in this review, 11 (18.3%) [18-28] were conducted in horses, and 49 (81.7%) [29-77] were conducted in humans. Considering both species together, the results of the clinical trials were evenly distributed, that is, beneficial effects were observed in 28 studies (46.7%) [19,21-23,25,26,28,29,32,34-36,38,39,41,49,55,57,58,62,64,69,70,72-76], and negative effects were observed in 26 studies (43.3%) [18,30,31,33,37,42-45,47,48,50-54,56,59,60,63,65-68,71,77] after PRP application (Additional file 1: Table S1).

Analyzing the species separately, we observed that the distribution of results was not homogeneous and that positive results were more frequent than negative results in the equine species. Seven [19,21-23,25,26,28] of 11 equine clinical trials yielded positive results (63.6%), three [20,24,27] resulted in partially positive results (27.3%), and one (9.1%) [18] yielded a negative result. In contrast, of the 49 human clinical studies, 21 yielded positive results (42.8%) [29,32,34-36,38,39,41,49,55,57,58,62,64,69,70,72-76], while 25 studies (51%) [30,31,33,37,42-45,47,48,50-54,56,59,60,63,65-68,71,77] yielded negative results, and 3 (6.1%) [40,46,61] resulted in partially positive results.

Although the percentages of negative and positive results in the human clinical trials were fairly even, the study design distribution among the positively and negatively evaluated studies was not homogeneous. This distribution revealed a negative correlation between a rigorous study design and the finding of beneficial results associated with the clinical use of PRP. Thirty-five studies were classified as RCTs: 34 [29,30,32-40,43,44,46,47,49-54,56,57,59,61-63,66-68,70,71,75,77] from the human medical literature and one [18] from the equine medicine literature. Among them, 12 trials (34.3%) [29,32,34-36,38,39,49,57,62,70,75] yielded positive results, while 20 RCTs (57.1%) [18,30,33,37,43,44,47,50-54,56,59,63,66-68,71,77] yielded negative results, and 3 RCTs (8.6%) had [40,46,61] mixed results.

Among the remaining 10 equine clinical trials that were not RCTs, seven studies [19,21-23,25,26,28] that yielded positive results were uncontrolled trials. Overall, 2 studies (18.2%) [18,24] had control groups, and eight [19,21-23,25-28] uncontrolled case series accounted for 72.7% of the equine studies. The remaining 15 human studies [31,41,42,45,48,55,58,60,64,65,69,72-74,76] that were not RCTs were controlled retrospective or prospective cohorts, comparative studies, case control series and observational controlled studies.

Additionally, while 76.9% (20/26) of the studies [18,30,33,37,43,44,47,50-54,56,59,63,66-68,71,77] with negative results were classified as RCTs, 42.8% (12/28) of the studies [29,32,34-36,38,39,49,57,62,70,75] with positive results were RCTs.

Regarding study design, the lack of a true placebo control group was the most frequently assigned flaw. Forty-three studies (71.7%) [24,29-32,35,38-44,46-55,57-76] had control groups that differed from placebo, such as hyaluronic acid and corticosteroids, and/or that included physiotherapy, excentric exercises, peppering techniques, dry needling (or combinations of these techniques) or merely lack of PRP application. Nine studies (15%) [19-23,25-28] lacked a control group, and all of these studies were conducted in horses.

Poor PRP characterization was a feature of 35 clinical studies (58.3%) [20,24,29-33,35-38,42-47,49,50,53,55-60,62-64,67,68,71,73,76,77] and was the second most frequent flaw. Information regarding PRP preparation, activation and composition was not always provided in a complete manner, and in one clinical trial [60], this information was not provided at all. The product name, a description of the processing method in detail and a description of PRP activation when used (or stated otherwise) defined adequate hemoderivative characterization. Platelet and leukocyte concentrations, together with their concentration factors from baseline levels and an analysis of growth factor contents, should ideally have been stated. Of 60 clinical trials, eight (13.3%) [21,22,25,27,28,51,69,72] provided this standard of information. Seventeen studies (28.3%) [18,19,23,26,34,39-41,48,52,54,61,65,66,70,74,75] provided only average-quality information on the employed hemoderivative; no data on growth factor content were provided.

The methods employed for PRP preparation have been the object of controversy [1,7]. Of the 60 clinical studies, 30 (50%) [18,19,23,29,31-36,38,39,46,51,53,54,56,57,62-64,67-70,72,73,75-77] employed only one centrifugation, 19 (31.7%) [21,24,25,27,28,42-45,47,49,50,52,55,58,59,65,66,71] employed two centrifugations, and one [41] employed three centrifugations. Filtration was the method of choice for obtaining PRP in one study [22], and nine studies [20,26,30,37,40,48,60,61,74] did not state how many centrifugations were employed for PRP preparation. Of the 30 studies that employed one centrifugation, 18 yielded positive results (60%) [19,23,29,32,34-36,38,39,57,62,64,69,70,72,73,75,76]. Of the 19 studies that employed 2 centrifugations, 6 yielded positive results (31.6%) [21,25,28,49,55,58].

Regarding PRP activation, 23 studies [18,23,24,29-33,35,37,38,41,47,51,53,54,56,57,60,62,63,76,77] did not stipulate whether their product was activated or not. Overall, 35 studies (58.3%) [19-21,25-28,33,36,39,40,42-46,48-50,52,55,58,59,61,65-75] used activators together with PRP. Calcium was used in 23 studies [19,21,25,27,28,33,36,39,42-45,48-50,52,55,58,59,65,66,69,72] thrombin in five [20,61,67,73,74], and calcium and thrombin in combination in six [40,46,68,71,73,74]. In two studies [22,64], the PRP was not activated.

Insufficient blinding was the third most common flaw in design and was found in 33 studies (55%) [18-31,35,37-41,45,48,55,58,60,65,67,69,72-76]. Adequate blinding was defined as the outcome assessors, patients and treating physicians being blinded to the treatment or intervention that was applied. Among the 33 non-blinded studies encountered in this review, 19 (57.6%) [19,21-23,25,26,28,29,35,38,39,41,55,58,69,72-74,76] had favorable results associated with PRP intervention, and ten (30.3%) [18,30,31,37,45,48,60,65,67,77] yielded negative results.

The fourth most frequent flaw, occurring in 32 studies [18-28,30,35,37,40,43,45,46,51,52,56,58,59,61,64,65,67,70,72,75-77] (53.3%), was the enrollment of a smaller-than-desired number of subjects. A study was considered to have a small sample when this information was provided by the respective authors, based on power calculations of the selected population. Among the 28 studies in which PRP had positive effects, 14 (50%) [19,21-23,25,26,28,35,58,64,70,72,75,76] had a small sample, and among the 26 studies in which PRP intervention resulted in negative outcomes, 12 (46.1%) [18,30,37,43,45,51,52,56,59,65,67,77] enrolled a smaller-than-desired sample.

The adoption of a short follow-up period, that is, an observation period of less than 12 months, also occurred in 32 studies (53.3%) [20,23,29,32-42,51,53-55,59-61,63,65,67-71,73-75,77]. Among the 26 clinical trials with negative results, 12 (46.1%) [18,30,31,43-45,47,48,50,52,56,66] were considered to have sufficient duration. Among the 28 studies with positive results, 13 (46.4%) [19,21,22,25,26,28,49,57,58,62,64,72,76] were considered to have an appropriate follow-up period.

Inadequacy of the parameters for outcome evaluation was a feature of 29 studies (48.3%) [18,24-26,28,29,31,32,34,36-39,41,42,51,54,55,57,61-64,67,69,70,74-76]. Among the 26 studies with negative results, 8 [18,31,37,42,51,54,63,67] adopted inadequate outcome measures (30.8%). Among the 28 studies with favorable results, 19 [25,26,28,29,32,34,36,38,39,41,55,57,62,64,69,70,74-76] had inadequate outcome measures (67.8%), and nine studies [19,21-23,35,49,58,72,73] were classified as having adequate outcome measures.

**Figure 2:**
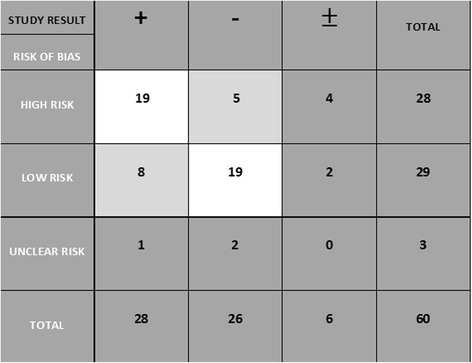
Association between risk of bias and clinical studies’ results.

Not only study design but also the evaluation of the risk of bias of individual studies showed relationships between studies with a higher risk of bias and positive (beneficial) results observed after PRP interventions, with 67.8% (19/28) [19,21-23,25,26,28,29,34,38,39,41,55,58,69,72-74,76] of high-risk studies having positive outcomes. Of the studies rated a low risk, 28.6% had favorable outcomes. Among all of the studies in which PRP did not positively affect the selected outcomes, 73.1% (19/26) [18,30,33,37,43,44,47,50-54,56,59,63,66,68,71,77] were attributed a low risk of bias (Table 1 and Figure 2).

**Table 1:** **Risk of bias of selected clinical studies**

	**Random sequence generation (selection bias)**	**Allocation concealment (selection bias)**	**Blinding of participants and personnel (performance bias)**	**Blinding of outcome assessment (detection bias) (patient-reported outcomes)**	**Blinding of outcome assessment (detection bias) (all-cause mortality)**	**Incomplete outcome data (attrition bias) (short-term [2-6 weeks])**	**Incomplete outcome data (attrition bias) (long-term[> 6 weeks])**	**Selective repoorting (reporting bias)**	**Risk of bias**
Garret 2013	+	+	-	+	-	+	+	-	**↓**
Zuffova 2013	-	-	-	-	-	+	+	+	**↑**
Edinger 2012	-	-	-	-	-	-	-	-	**↑**
Torricelli 2011	-	-	-	-	-	+	+	+	**↑**
Castelijns 2011	-	-	-	-	-	+	+	+	**↑**
Georg 2010	-	-	-	-	-	+	**?**	-	**↑**
Abelanet 2009	-	-	-	-	-	+	+	-	**↑**
Carmona 2009	-	-	-	-	-	+	+	+	**↑**
Waselau 2008	-	-	-	-	-	+	+	+	**↑**
Arguelles 2008	-	-	-	-	-	+	+	+	**↑**
Carmona 2007	-	-	-	-	-	+	+	+	**↑**
Tiwari 2013	**?**	-	-	-	**?**	+	+	-	**↑**
Antuna 2013	+	+	-	+	+	+	+	+	**↓**
Magnussen 2013	-	-	-	-	-	+	+	+	**↑**
Mishra 2014	+	+	-	+	+	+	+	+	**↓**
Krogh 2013	+	+	-	+	+	+	+	+	**↓**
Patel 2013	+	**?**	**?**	**?**	**?**	+	+	-	**↑**
Wasterlain 2012	+	+	-	+	+	+	+	+	**↓**
Jain 2012	+	+	+	+	+	+	+	+	**↓**
Mardones 2012	+	+	+	+	+	+	+	+	**↓**
Cerza 2012	-	-	-	-	-	+	+	+	**↑**
Mei-Dan 2012	+	-	-	-	+	+	+	+	**↑**
Almeida 2012	+	-	-	-	+	+	+	+	**↓**
Spaková 2012	-	-	-	-	-	+	+	+	**↑**
Aksahin 2012	-	-	-	+	-	+	+	+	**?**
Rodeo 2012	+	+	+	+	+	+	+	+	**↓**
Weber 2012	+	-	+	+	+	+	+	+	**↓**
Bergeson 2012	-	-	-	-	+	+	+	+	**?**
Cervelin 2012	+	+	**?**	-	+	+	+	-	**↓**
Filardo 2012	+	+	+	+	+	+	-	+	**↓**
Jo 2011	-	-	-	-	-	+	+	+	**↑**
Randelli 2011	+	+	+	+	+	+	+	+	**↓**
Castricini 2011	+	+	+	+	+	+	+	+	**↓**
Thanasas 2011	+	+	-	+	+	+	+	+	**↓**
Schepul 2011	+	+	+	+	+	+	+	+	**↓**
De Vos 2011	+	+	+	+	+	+	+	+	**↓**
Creaney 2011	+	+	+	+	+	+	+	+	**↓**
Kon 2011	-	-	-	-	-	+	+	+	**↑**
Jonge 2011	+	+	+	+	+	+	-	+	**↓**
Gosens 2011	**?**	**?**	+	+	+	+	+	+	**↓**
Barber 2011	-	-	-	-	-	+	+	+	**↑**
Horstman 2011	+	+	+	+	+	+	+	+	**↓**
Buford 2011	-	-	-	-	-	+	+	+	**↑**
Vogrin 2010	+	+	+	+	+	+	+	+	**↓**
Peerboms 2010	+	+	+	+	+	+	+	+	**↓**
De Vos 2010	+	+	+	+	+	+	+	+	**↓**
Radice 2010	-	-	-	-	+	+	+	+	**?**
Filardo 2010	-	-	-	-	-	+	+	-	**↑**
Nin 2009	+	+	+	+	+	+	+	+	**↓**
Silva 2009	-	-	-	-	-	+	+	+	**↑**
Peerboms 2009	**?**	+	+	+	+	+	-	+	**↓**
Sanchez 2008	-	-	-	-	-	-	**?**	+	**↑**
Everts 2008	+	+	+	+	+	+	+	+	**↓**
Orrego 2008	+	+	-	+	+	+	+	+	**↓**
Sanchez 2007	-	-	-	-	-	+	+	+	**↑**
Everts 2007	-	-	-	-	-	+	+	+	**↑**
Gardner 2007	-	-	-	-	-	+	+	+	**↑**
Zavadil 2007	+	+	+	+	+	+	+	+	**↓**
Mishra 2006	-	-	-	-	-	+	-	+	**↑**
Ventura 2005	+	+	-	-	-	+	+	+	**↓**

Side effects associated with PRP use in the clinical setting were observed in two studies (3.3%); in one study [18] they referred to swelling at the injection site and in another [45] they related to infection after platelet-rich fibrin matrix application.

The majority of *in vivo* trials were devoted to observing the effects of PRP on tendinopathies (29 studies or 46%) [19-21,24,27,30,32,33,35,40,43-45,48-54,56-58,60,62,-63,65,72,76], followed by articular disorders (18 studies or 28.6%) [24,25,28,34,36-39,41,47,55,59,68-70,73-75] and ligament disorders (16 studies or 25.4%) [18,21-23,26,27,29,31,42,46,61,64,66,67,71,77]. The sum of recorded lesion locations (63 injury sites) exceeded the total number of clinical articles selected because a few studies observed the effects of PRP on both tendons and ligaments [21,23,27]. Considering 63 observation points, the injured tissues that were more positively affected by PRP treatment in *in vivo* trials were cartilage (20.6%) [25,28,34,36,38,39,41,55,69,70,73-75] and tendons (17.5%) [19,21,23,32,35,49,57,58,62,72,76] followed by ligaments (9.5%) [21-23,26,29,64]. Among the studies of arthropathies, 72.2% yielded positive results. In addition, 37.9% of the studies of tendinopathies yielded positive results, and 37.5% of the studies of desmopathies yielded positive results.

### Experimental studies with PRP

In total, 63 experimental studies were included in this review: 13 (20.6%) [78-90] were conducted in horses, 16 (25.4%) [91-106] were conducted in humans and 34 (54%) [107-140] were conducted in other species.

Overall, their results contrasted with those of the clinical trials. Regarding outcomes, the homogeneity of the distribution between beneficial and negative results observed in the clinical trials was not mirrored in the experimental scenario. Among the 63 experimental studies, 46 (73%) [81-86,88,90-95,97-103,105-110,112,115,118-122,124-129,131,134-137,139,140] yielded positive results, 12 (19%) [78,79,87,89,96,104,111,113,116,123,130,133] yielded mixed results, and five (7.9%) [80,114,117,132,138] yielded negative results. Among the five experimental studies with negative results, all of them were conducted *in vivo;* four of them included mechanical evaluations among their outcome measures [80,117,132,138]. Two studies [114,132] exhibited side effects associated with PRP administration, and one study [114] failed to show improvements during the histological evaluation of experimentally induced tendon lesions treated with PRP.

From the 63 experimental studies, 35 (55.5%) [79,80,82-85,90,107,109-114,116-121,123-128,130-133,135-139] originated from *in vivo* experiments, 23 (36,5%) [81,86-89,91-103,105,108,115,133,139] originated from *in vitro* work, and five (7.9%) [78,96,104,106,122] originated from both *in vivo* and *in vitro* experiments (Additional file 2: Table S2). Considering separately the results of the five experiments conducted both *in vivo* and *in vitro* there are 67 experimental results. From the 41 *in vivo* trials, 27 (65.8%) [82-85,90,96,106,107,109,110,112,118-122,124-130,134,136,137,139] yielded positive results, while 23 of the 26 (88.5%) [81,86,88,91-103,105,106,108,115,122,134,140] *in vitro* experiments had positive results.

Considering only equine and human studies, there were 23 (79.3%) [81-86,88,90-103,105,106] positive results, 5 (17.2%) [78,79,87,89,104] mixed results and one (3.4%) [80] negative result.

Among the 12 experiments with partially positive results, nine (75%) [78,79,96,111,113,116,123,130,133] were conducted *in vivo.* Different reasons for adverse outcomes were observed. In one experiment [78], the PRP preparation resulted in an insufficient increase in growth factor content. In two experiments [78,79], side effects related to PRP administration were observed. In another two experiments [111,130], PRP treatment did not result in hyaline cartilage formation. In four studies [113,116,123,133], the treated tissues did not satisfactorily withstood mechanical challenges and in one trial [96], gene expression was not affected by PRP. In two *in vitro* trials with partially positive results [87,89], equine suspensory ligament explants cultured with PRP had increased gene expression, but this rate was greater when acellular bone marrow was added to the cultures, and in one [78] PRP preparation resulted in an insufficient increase in growth factor content.

In total, side effects were observed in five experiments [78,79,114,117,132]. One study [114] observed local reactions at different injection sites and concluded that PRP was capable of initiating an inflammatory response in the absence of an injury. In a second trial [117], a cellular response around grafts for anterior cruciate ligament (ACL) reconstruction was noted. In two studies [78,79], thrombin-activated PRP elicited both a local and a systemic inflammatory response after intra-articular injections in horses. Others (132) noticed cellular infiltration and fibroid necrosis 7 days after PRP application in a rotator cuff repair model.

Regarding PRP characterization, 16/63 (25.4%) experiments [78,79,81,87,88,90,98,100-103,105,113,125,135,140] exhibited good product characterization; in 26 studies (41.3%) [82,85,86,92-94,96,97,99,104,106-108,111,112,114,116,117,122,126,128-130,134,136,137] the hemoderivative was satisfactorily described, and in 21 (33.3%) [80,83,84,89,91,95,109,110,115,118-121,123,124,127,131-133,138, 139] experiments, the characterization was considered unsatisfactory.

Activation was employed in 39 experiments (61.9%) [78,79,82,85,92,94,96-108,110,112-114,116,117,119-122,125-127,129,130,132,134,136,137,140], and PRP activation was not mentioned in 23 experiments (36.5%) [80,81,83,84,86-91,93,95,109,111,115,118,123,124,130,133,135,138,139]. One study [128] stated that the hemoderivative was not activated.

For the preparation of PRP in experimental studies a single centrifugation was the method chosen for PRP preparation in 29 experiments (46%) [81,83,84,86-91,94,97,98,100,103-105,109-111,113,118-121,124,125,128,133,139]. Two centrifugations were used in 29 (46%) [80,82,92,95,96,99,101,106-108,112,114-117,122,123,126,127,129-132,134-138,140] experiments, one and two centrifugations were used in one experiment [93] and 3 centrifugations were employed in two experiments [85,102]. Filtration was used to obtain PRP in two trials [78,79]. Among the 29 experiments that employed one centrifugation, 25 yielded positive results (86.2%) [81,83,84,86,88,90,91,94,97,98,100,102,103,105,109,110,118-121,124,125,127,139,140]. Of the 29 experiments that employed two centrifugations, 20 yielded positive results (75%) [82,92,94-96,99,101,106-108,112,115,122,127,131,134-137,140].

Among the 67 experimental results, 17 were conducted in humans [91-106], 15 in rabbits [113-127], 13 in horses [78-90], ten in rats [128-137], five in sheep [96,104,110-112], three in pigs [138-140], three in dogs [107-109] and one in mice [96]. The number of species exceeds the number of experiments because, in a few cases, *in vivo* and *in vitro* trials of a same experiment yield two results in the species count down.

Thirty-five experiments (55.5%) [80,81,83-85,88,90-97,102,104-106,108,110,116,119-121,124,125,128,129,131-137] were related to tendon disorders, 16 (25.4%) [78,79,82,98-101,103,111,112,115,123,126,127,130,140] to cartilage, ten (15.9%) [87,89,107,109,113,117,118,122,138,139] to ligament lesions, one (1.6%) [86] to both ligament and tendon disorders, and one (1.6%) [114] to several potential injury tissues. PRP yielded positive results in 85.7% of the experiments with tendons (30 trials) [81,83-85,88,90-95,97,102,104-106,108,110,119-121,123,124,128,129,131,134-137], 68.7% (11 trials) of those with cartilage [82,98-101,103,112,115,126,127,140], and 50% of those with ligaments (five trials) [107,109,118,122,139].

## Discussion

The use of hemoderivatives for tissue healing has gained increasing popularity for the treatment of musculoskeletal lesions. Among these derivatives, PRP has already been established as a part of the repertoire of possibilities for the treatment of orthopedic conditions [4,6,10,141-147].

Despite widespread acceptance of its ambulatory use, research continues for the purpose of providing convincing evidence of clinical benefits associated with this hemoderivative’s administration. Uncontrolled or biased reports of PRP’s efficacy have been excessive and have not strengthened the existing evidence, suggesting (but not definitively demonstrating) the beneficial effects of PRP.

The overall quality of the study design was less than ideal in the majority of the selected studies and the quality was inversely correlated with the performance of PRP in clinical trials. Rigorous study design and a low risk of bias were associated with negative outcomes in PRP clinical trials. Because a high-quality study design was a much more common feature of human studies and a rare feature of equine studies, it was not surprising to find more negatively affected outcomes in human clinical trials and positive outcomes in equine clinical trials. Other authors have emphasized the importance of well-designed clinical studies for the evaluation of PRP’s efficacy and limitations and these authors have warned about their scarcity [141,146,148-156], but the present review presented a quantitative link between study design and outcome.

Accordingly, 76.9% of the studies with negative results were classified as RCTs, while RCTs comprised only 42.8% of the studies with positive results. While the inclusion of studies with different designs can be a subject to debate if, in one hand, the inclusion of RCTs, cohorts and controlled case series caused heterogeneity of our study sample, on the other, the diversity and the large number of studies included allowed for comparisons and for the establishment of a quantitative relationship between study design and outcome after PRP intervention. This relationship was more consistent in the human clinical trials, given the scarcity of RCTs and controlled studies in the equine species. As expected, the majority of less than ideally- designed equine studies yielded positive results, and the only negative result among equine clinical trials originated from the only equine RCT. The same was true for including data of clinical and experimental studies, but, again, the comparison between results of PRP´s efficacy with these different methodologies revealed important results.

Another purpose of the current review was to analyze and summarize the most common flaws in study design and to evaluate their implications in the results of PRP interventions. The most striking feature contributing to the debatable quality of the clinical trials included in this review was the lack of a true placebo control group. This observation referred mainly to the human clinical trials because the majority of the equine clinical trials lacked a control group, thereby preventing comparison.

Because several of the selected studies adopted subjective outcome measures and lacked a true placebo control group, their results could have been impacted. However, a gold standard treatment, if one exists, can be assigned to the control group for comparison with a new proposed treatment. Then, researchers would be comparing two active treatment groups, without a placebo group [157]. This comparison was frequently observed in this review, and PRP’s effectiveness was often compared with that of hyaluronic acid, corticosteroids, autologous blood injections, no treatment, dry needling, physiotherapy or combinations of these therapies. Nevertheless, treatment controls, such as sham acupuncture and intra-articular hyaluronic acid, have a greater effect size than the average placebo effect [158]. Although control groups were often assigned to some sort of treatment in the selected articles, we could not identify relationships between positive or negative results and the lack of a true placebo group. In addition, as other authors [159] have mentioned, the use of PRP combined with other biological therapies could pose a challenge to the evaluation of PRP’s individual effects and confound interpretation of the results. Therefore, studies that chose to do so were not included in this review.

Poor hemoderivative characterization was the second most common flaw contributing to the assignment of study design as not ideal. Inconsistencies associated with PRP preparation and administration have contributed to the lack of strength and to the disparities of the generated evidence regarding PRP’s efficacy, in both clinical and experimental studies [160]. The most important consequence of the confounding diversity of the methods employed for PRP processing was the difference in resulting final products, which precluded a comparison of the treatment’s results [143,149,156]. Similar to any autologous blood-derived product, PRP has unique, non-reproducible and donor-related features that can jeopardize a comparison of the results [161,162]. Therefore, the hemoderivate composition should be verified and clearly presented [81,163] to minimize these effects.

This review confirmed the existing diversity of preparation methods, commercial or laboratorial, for obtaining PRP, as already indicated by other researchers [12,81,149,156,164-166]. These methods might include single, double or triple centrifugations, filtration and plateletpheresis, with and without the aid of activating agents. Most of the selected clinical studies employed one centrifugation step, and more positive results were observed with this method. Previous studies have demonstrated that a double centrifugation method resulted in higher platelet concentrations [167,168], but caused more alterations in platelet morphology and was more sensitive to small errors during preparation [168,169] compared with the single centrifugation method. This trend toward more positive outcomes with PRP prepared with one centrifugation step must be confirmed with further research. Overall, PRP characterization was more adequate in the experimental studies than in the clinical trials.

Another controversial topic in PRP preparation has been the need for activation – or the lack of it [163,170-172]. Until a consensus is reached, comparing activated to non-activated products is inevitable. Most of the clinical and experimental studies activated PRP before injection, and clinically more positive outcomes were observed without PRP activation. The percentages of clinical and experimental studies that employed activation were similar. Experimentally, there was no relationship between the use of activation before PRP administration and favorable or unfavorable outcomes.

The administration protocols also varied greatly among the studies - clinical and experimental - regarding injection or application techniques, volume of hemoderivative employed and timing and frequency of administration. Again, all of these variables limited our ability to compare the results from different articles [148,166].

Poor blinding was the third most frequent negative feature of the studies designs. Blinding limits bias in outcome evaluation, and whenever feasible, outcome assessors should not be aware of the treatment allocations of the patients in a clinical study [173]. Blinding of outcome assessors is one of the safeguards to assure the internal validity of a trial, and there has been strong evidence that their unblinding exaggerated treatment effects. Particularly when scoring subjective outcomes, for instance pain scores, biased findings can result from inadequate blinding [157,174]. Blinding becomes less important in reducing observer bias, as the outcome measure becomes less subjective [157]. In addition to the blinding of outcome assessors, trial participants and investigators should be unaware of an assigned intervention for similar reasons.

The adoption of a short follow-up period and the enrollment of a small sample were the fourth most commonly encountered weaknesses in study design, particularly in horses. Inadequate duration of follow-up or treatment compromises the external validity of an RCT. Clinicians treating patients with a variety of conditions have called attention to the contrast between the beneficial effects of treatments in short-term RCTs and the less encouraging experiences with long-term treatment in clinical practice [175]. Long-term follow-up evaluations should be a priority [81]. In this review, the classification of short study duration was evenly distributed between studies with positive and negative results and did not particularly affect outcomes, although a few clinical trials showed the short-term efficacy of PRP in improving knee function and quality of life [55,176].

The detrimental effects of enrolling a small sample on the power of a study have been well documented. When a sample is too small, a study is particularly susceptible to a type II errors; that is, the study could be insufficiently powerful to detect real differences between observed groups. Calculations of explicit sample size or power to anticipate this error have rarely been performed before the start of a research study [177]. However, in the selected articles, smaller samples were not particularly associated with positive or negative outcomes. Nevertheless, we encourage authors to enroll sufficient numbers of subjects in their studies to assure adequate power and significance to their findings.

The adoption of inadequate outcome measures was the fifth most frequently encountered weakness in study construction in this review. As already mentioned subjective outcomes present great opportunities for bias [174] and frequently were the only type of employed outcome measure in the selected studies.

In orthopedic research, health status can be assessed by a number of methods, which are classified either as objective (e.g., radiological changes, range of motion) or as subjective (those relying on responses obtained directly from patients about their perceptions of health and illness) and as either generic or disease-specific [174]. Most authors have accepted that a combination of objective and subjective measures is desirable for conducting a complete assessment. Often, a single given parameter employed for outcome evaluation suits certain circumstances but not others, and there has rarely been a single most appropriate rating system or outcome measure [173,178,179]. Employing different outcome measures allows for the capture of diverse aspects of overall function, and lack of agreement between patient-reported and objective measures reflects this diversity, rather than indicating a weakness in one method or the other [180].

In this review, 48.3% of the selected studies only evaluated PRP treatment’s efficacy with a few questionnaire-reported or subjective measures, as opposed to the ideal multifaceted evaluation; therefore, they were classified as having poor outcome measures. It is important to choose the most adequate measures for a particular task, condition and setting; otherwise, the results of clinical research can be misleading. In particular, the inclusion of mechanical tests resulted in more outcomes that were negative in the experimental studies. This association might help to explain why the consistently positive responses induced by PRP *in vitro*, particularly in cell cultures, were not correlated with similar improvements in outcomes *in vivo*. Another aspect that added confusion to the comparison of results of PRP treatments among the studies was the variety of lesions treated. PRP’s performance was compared in acute and chronic; experimentally induced and naturally occurring; tendon, ligament and articular disorders without due distinction for the particular tissues’ characteristics. Biomechanical particularities, such as those of the flexor and extensor tendons; lesion localization, such as insertional versus body tears in ligaments; and staging of lesions, such as advanced osteoarthritis versus mild cartilage injuries, were not considered when evaluating PRP’s treatment efficacy [166,177].

The localization of lesions in the clinical trials was associated with both positive and negative outcomes. PRP intervention yielded more positive results in tendinopathies than in arthropathies and desmopathies in humans. In a large survey of PRP’s effects in the equine species in which 191 subjects were treated for desmopathies, tendinopathies and arthropathies, good results after intralesional injection were obtained, but in tendinopathies results were more impressive [181]. The reasons for these findings are unknown, and given the heterogeneity of tissues and stage of diseases, these results should be interpreted with care. Other researchers failed to provide definite recommendations for PRP intervention on a tissue-related basis [164]. Clinical and experimental scenarios, as well as interventions in distinct phases of lesion progression, different species and particularly-affected structures do affect performance of a treatment modality and must be considered.

This review demonstrated that scientific evidence of the beneficial effects of PRP in clinical settings remains lacking. While the vast majority of experimental *in vitro* studies yielded positive results, the same promising outcomes were not verified in the clinical trials after intervention with PRP, particularly in humans. Corroborating these observations, we found that all of the negative results from experimental works came from *in vivo* studies. Other authors have made similar remarks when comparing *in vivo* and *in vitro* studies of PRP [78,182-184], but the present review furnished numeric data for making comparisons and drawing conclusions. Altogether, these numbers demonstrated that the high expectations created by PRP’s outstanding performance when tested *in vitro* have not been fulfilled when studies have been performed in living subjects. Given the small number of experimental studies with negative results, factors that could have adversely affected the outcomes were searched. Among the experimental *in vivo* studies that yielded negative results, four included mechanical evaluations in their outcome measures. Two studies exhibited side effects associated with PRP administration, and one study failed to show improvements during the histological evaluation of experimentally induced tendon lesions treated with PRP. The trend toward failure when mechanical tests were applied in PRP-treated tissues was already noted in *in vivo* studies with mixed results and was more evident when the follow-up period lasted longer after the PRP intervention. Mechanical tests should be considered when testing PRP’s effects in experimental models.

Why *in vitro* positive results outpaced the positive results observed in clinical and *in vivo* PRP studies by such a large margin remains to be determined. The particular characteristics of these distinct environments may provide clues about this discrepancy. The *in vivo* environment presents several variables that are absent outside of a living organism and that could interfere with PRP’s effectiveness. However, the primary advantage of *in vitro* research is that it allows for an enormous level of simplification of the system under study and of the effects of a compound on a system. As a drawback, *in vitro* experiments fail to replicate the precise cellular conditions of a living organism. They are conducted in a closed system that tend to allow for higher and longer exposure of cells or explants to a given molecule than the exposure found in body tissues, which are half open systems. *In vivo*, substances cannot easily reach target cells and are subject to biokinetics, which can result in underestimation of their effects [185].

Another relevant aspect that could harm the extrapolation of *in vitro* results to the *in vivo* scenario was the lack of consideration of interspecies differences. Numerous experimental *in vivo* studies have enrolled different animal models, and this finding could further affect the interpretation of the results of the efficacy of PRP in horses and humans.

Overall, PRP has proved to be a safe therapeutic tool, with few adverse effects observed in the selected articles. This finding was similar to reports by other authors [55,164,177,181,186]. Although the *in vitro* antibacterial effect of human PRP against methicillin-resistant *Staphylococcus aureus* has been demonstrated [187], as well as those of equine platelet concentrates and platelet-poor plasma [188] the potential risk for bacterial contamination during PRP processing must be considered.

## Conclusion

PRP has demonstrated the potential to exert beneficial effects in the healing of tendons, ligaments and cartilage, but definitive clinical evidence of its efficacy remains lacking. A great amount of literature has been dedicated to the topic, and well-constructed clinical trials, with sufficient power and duration to detect differences between treatments, as well as standardization of products, procedures and conditions to be treated, are as important as they are scarce.

Our results confirmed that biased, poorly designed studies, that are not properly controlled or blinded and that adopt inadequate outcome measures, favored the observation of positive results. As a consequence of this finding, the majority of equine clinical studies, which lacked randomization; blinding; and adequate power, outcome measures and control groups, yielded positive results. Similarly, human clinical trials with analogous undesirable features tended toward positive outcomes. Although the human clinical trials were better constructed than the equine studies, some aspects of their design must nevertheless be improved to generate strong evidence regarding the use of PRP in clinical scenarios.

## Additional file

Additional file 1: Table S1. Characteristics of 60 clinical studies that provided evidence regarding PRP intervention. Additional file 2: Table S2. Characteristics of 63 experimental studies that provided evidence regarding PRP intervention.

## Abbreviations

ABI: Autologous Blood Injection
ABM: Acellular Bone Marrow
ACL: Anterior cruciate ligament
ACP: Autologous Conditioned Plasma, ADAMTS, A desintegrin and metalloproteinase with thrombospondin motifs
AHFS: Ankle Hindfoot Scale
AOFAS: American Orthopaedic Foot and Ankle Society
ASES: American Shoulder and Elbow Surgeons
ATRS: Achilles Tendon Total Rupture Score
bFGF: basic fibroblast growth factor
BMA: Bone marrow aspirate
BMMC: Bone marrow mononucleated cell
BMP: Bone morphogenetic protein
BMS: Betamethasone
Centrif.: Centrifugation
Ca: Calcium
ChM-I: Chondromodulin-I
COL: Collagen
COMP: Cartilage oligomeric matrix protein
COX-2: Ciclo oxigenase- 2
CSA: Cross sectional area
CT: Computed tomography
CXCR4: C-X-C chemokine receptor type 4
DASH: Disabilities of the Arm, Shoulder and Hand
DDFT: Deep Digital Flexor Tendon
DMEM: Dulbecco’s modified eagle medium
DS: Double spin
EA: Extraarticular
EGF: Endothelial growth factor
ES: Echogenicity score
Ex: Examination
FAS: Fiber alignement score
FBS: Foetal bovine serum
FCS: Foetal calf serum
FGF: Fibroblast growth factor
GAG: Glycosaminoglycan
GAPDH: Glyceraldehyde 3- phosphate dehydrogenase
GF: Growth factor
HA: Hyaluronic acid
Hb: Haemoglobin
HDGF: Hepatocyte-derived Growth Factor
hGH: human growth hormone
HMW: High molecular weight
HS: Human serum
IA: Intraarticular
ICL: Inferior Check Ligament
ICRS: International Cartilage Repair Society
IGF-1: Insulin growth factor-1
IKDC: International Knee Documentation Committee
IL-1: Interleukin-1
IL-6: Interleukin-6
Inj: injection
KOA: Knee osteoarthritis
KOOS: Knee Injury and Osteoarthritis Outcome Score
KSS: Knee society score
KT-1000: KT-1000 arthrometer
Le: Leukocyte
LMW: Low molecular weight
MFx: Microfractures
MIZ: Maximum injury zone
MMP: Metalloproteinase
MPC: Mean platelet count
MPD: Methylprednisolone
MRI: Magnetic resonance imaging
NFκB: Nuclear factor kappa B
NRS: Numeric Rating Scale
OA: Osteoarthritis
OA*: Outcome acessors
OC: Osteochondrosis
P: Patient
PBS: Phosphate buffered saline
PC: Platelet concentrate
PCR: Polimerase chain reaction
PDGF: Platelet-derived growth factor
Pl: Platelet
PP: Platelet-poor
PPACD: Platelet product acid citrate dextrose
PPCPD: Platelet product citrate phosphate dextrose
PPCR: Platelet-poor clot releasate
PRCR: Platelet-rich clot releasate
PPPR: Platelet-poor plasma releasate
PRF: Platelet-rich fibrin
PRFM: Platelet-rich fibrin matrix
PRGF: Platelet-rich growth factor
PRP: Platelet-rich plasma
PRPR: Platelet-rich plasma releasate
PRTEE: Patient-rated Tennis Elbow Evaluation
PSD: Proximal suspensory desmitis
PTGE: Prostaglandin endoperoxide synthase 2
RBC: Red blood cell
RCT: Randomized clinical trial
ROM: Range of motion
RTPCR: Reverse transcriptase polymerase chain reaction
SANE: Single Assessment Numeric Evaluation
SDFT: Superficial Digital Flexor Tendon
SER: Strength in External Rotation
SC: Subcutaneous
SF: Synovial fluid
Signif: Significant
SOX-9: Transcription factor SOX 9
SPADI: Shoulder Pain and Disability Index
SS: Single spin
SST: Simple Shoulder Test
T: Thrombin
TA: Triamcinolone
TGFβ-1: Transforming Growth Factor Beta-1
TIMP: Tissue inhibitor metalloproteinase
TNF-α: Tumor necrosis factor alpha
TP: Treating physician
UCLA: University of California - Los Angeles
US: Ultrasound
UTC: Ultrasonographic tissue characterization
VAS: Visual Analogue Score
VCAM: Vascular cell adhesion protein 1
VEGF: Vascular endothelial growth factor
VISA: Victorian Institute of Sports Assessment
WBC: White blood cells
WOMAC: Western Ontario and McMaster Universities Arthritis Index
WORC: Western Ontario Rotator Cuff
