# Supplementary material for: Platelet-rich plasma in orthopedic therapy: a comparative systematic review of clinical and experimental data in equine and human musculoskeletal lesions
Source: BMC Vet Res. 2015 Apr 22;11:98. doi: 10.1186/s12917-015-0403-z (PMC4449579; doi:10.1186/s12917-015-0403-z)
Supplement: Additional file 1: Table S1. — Characteristics of 60 clinical studies that provided evidence regarding PRP intervention. [file 12917_2015_403_MOESM1_ESM.doc]

**Additional file 1: Table S1**

| Authors/ year | Study design (Level of evidence)/ population | Follow up | Control | Outcome measures | Lesion | HEmoderivates obtention/ citology/ activation/ analysis | Intervention | Results | Blinding | OBS | Prp effect |
| --- | --- | --- | --- | --- | --- | --- | --- | --- | --- | --- | --- |
| Garret et al. 2013 [18] | RCT (II) Thoroughbred Yearlings n = 39 | 2 years | Saline injection (n = 19) | Racing performance/ money earned/ number of races | Proximal Sesamoid bone Inflammation + SL branch desmitis | GPS® II Separation kit Biomet^1^/ 1 centrif./ MPC = 966000 ± 189000/[Pl] ↑5,2X | 3.0 ml PRP inj. without US guidance (n = 20) | PRP treated horses were signif. more likely to start 1 race at 2 years of age. No signif. difference at 3 and 4 year-old racing years nor between groups regarding earnings | --- | Small N Scarcity of outcome measures Swelling at injection site in 6/20 treated horses No blinding | (−) |
| Zuffova; Krisova; Zert 2013 [19] | CASE SERIES (IV) n = 22 racing Thoroughbreds horses | 1 year | --- | Time to first race start, number of starts regarding lesion severity and stage of healing | SDFT tendonitis | Sedimentation + pressure extraction +1 centrif. /CaCl_2_ / [Pl] MPC = 466,5 x 10^9^/l / [Pl] ↑5,6x | US guided PRP inj. | Positive influence of PRP in acute and chronic SDF equine tendonitis | --- | Poor study design Small N | (+) |
| Edinger et al. 2012 [20] | CASE REPORT (IV) 1 equine | 13 weeks | --- | US, lameness evaluation | DDFT tendonitis within tarsal sheath | Thrombin | I) 2.0 ml PRP intralesional II) PRP + BMMCs intralesional - 13 weeks after | Clear US improvement after second treatment (8 weeks after),but lameness still present | --- | Poor study design, not blinded, not controlled Short follow up Small N Poor PRP characterization | (±) |
| Torricelli et al. 2011 [21] | CASE SERIES (IV) n = 13 equine athletes | 12 months | --- | US, clinical examination (lameness), athletic performance at 48 hours, 1 weekly, 12 months | Suspensory ligament desmitis or SDFT injury | 2 centrif. / MPC = 751 X 10^3^/μl 5,4x ↑ / CaCl_2_/ Mean GF: TGFβ1 3055, PDGFAB 357,1, VEGF 169,1, IGF 289,2, IL1β 3,9, EGF 4,6 | PRP + BMMNC intralesional 1 inj. US guided (4–7.0 ml) | 84.6% returned to competition All had US remission Fast recovery with platelet count › 750 X10^3^/μl in PRP | --- | Poor study design Small N No blinding No control | (+) |
| Castelijns et al. 2011 [22] | CASE SERIES / n = 11 horses | 1-3 years | --- | Clinical and ultrasonographic evaluation at 3 months | Acute suspensory ligament branch desmitis | E-PET System^10^ / MPC = 850 (±244) X 10^9^ / l [Pl] ↑7 X [Le] ↑5,2X [PDGF-BB] = 22,9 μg/ml | 2.5 ml PRP injected under US guidance in SL branch injuries (n = 18) | Clinical and ultrasonographic resolution in all cases; ↓ in lameness scores in 10/11 horses at 3 months Ultrasonographic resolution of lesions in 10/11 horses at 3 months 5/11 horses returned to previous level of performance, 3 were retired, 1 was exercising at lower level, 1 died | --- | Poor study design Small N No blinding No control No randomization | (+) |
| Georg et al. 2010 [23] | CASE SERIES (IV) n = 7 horses | 10 - 13 months | --- | Clinical and US evaluation (MIZ, CSA, ES, FAS) | SDFT = 6 limbs ICL = 1 limb DDFT = 1limb | ARTHREX®^5^ / 1 centrif./[PL] 1,3 X↑ /  MPC = 160-197 X 10^9^ / L [WBC] = 10,92 X↓ | 2-4.0 ml PRP intralesional, US guided | Clinical improvement and return to previuous level of performance without reinjuries report | --- | Poor study design Small N Short follow up No control No blinding | (+) |
| Abelanet; Prades 2009 [24] | CONTROLLED CASE SERIES n = 42 sport horses | 12 - 42 months | Chronic OA patients, unresponsive to rest or IA steroid therapy (n = 12) | Return to athletic performance rate of reinjury | Chronic (n = 20) and acute (n = 10) cases of OA | Manual double centrif. | 3 PRP injections (n = 30) | No signif. differences between PRP and control group in return to athletic performance. PRP treated horses had lower rate of reinjury. | --- | Small N Scarcity of outcome measures Group heterogenicity No randomization Not blinded No placebo control Poor PRP characterization | (±) |
| Carmona; López; Prades 2009 [25] | CASE SERIES n = 7 horses | 12 months | ---- | Degree of lameness and joint effusion | Severe joint disease (OA = 4 and OC = 3) | 2 centrif./ MPC = 259 X10^6^ /ml [Le]mean: 8,68 X 10^6^ /ml / CaCl_2_ [TGF-β1] mean: 12,5 ng/ml | 3 PRP inj. with 2 week-interval | Improvement in lameness degree and joint effusion in PRP, more so after last treatment | --- | Poor study design Small heterogeneous population No blinding No control Only clinical evaluation | (+) |
| Waselau et al. 2008 [26] | CASE SERIES (IV) standardbred race horses n = 9 | 3 years | --- | Race records (return to racing, number of starts, total earnings, earnings per start) | Moderate to severe midbody suspensory ligament desmitis | Buffy Coat Method / MPC = 1,37x10^6^/μl / Bovine thrombin | 1 US guided PRP inj. + gradual return to exercise | Treated horses had an excellent prognosis for returning to racing | --- | Small N No control No blinding Few outcome measures Poor study design | (+) |
| Argüelles et al. 2008 [27] | CASE SERIES (IV) n = 5 horses | 20 months | --- | Clinical evaluation and US evaluation (2, 12 and 20 months) | 2 SDFT tendonitis, 3 ChronicPSDs | 2centrif. / CaCl_2_ / MPC = 250 ± 71,8 x 10^6^, TGFβ1 12,515 ± 2,4 mg/ml | 4 intralesional PC inj. every 15 days (5–8.0 ml) | Positive effect of PRP in SDFT tendonitis. Horses with PSD did not show ultrasonographyc improvement in spite of clinical improvement. All horses returned to previous level of training | --- | Small N Poor study design Heterogeinicity of lesion treated Low platelet count on PC | (±) |
| Carmona et al. 2007 [28] | CASE SERIES (IV) n = 4 horses | 1 year | --- | Degree of lameness, US, SF analysis, joint effusion and clinical evaluation | Osteoarthritis at least of 1 year duration | 2 centrif./ MPC = 250 ± 71,8 x 10^6^,[Le] = 8,68 ± 3,78 x 10^6^/ CaCl_2_/ TGFβ1 = 12,515 ± 2,4 mg/ml / TP in synovial fluid | 3 inj. PC at 2 week-intervals | Signif. improvement in lameness degree and joint effusion, more so at 2 months after 3^rd^ treatment. All patients mantained improvements in lameness scores for about 8 months after last inj., then showed gradual deterioration | --- | Small N Poor study design No blinding Heterogeinicity of lesion treated No US results reported Low platelet count on PC | (+) |
| Tiwari; Bhargava 2013 [29] | RCT (I)/n = 60 | 6 months | Steroid (MPA 40 mg/ml) + 2% xylocaine (n = 30) | VAS at 1, 3 and 6 months | Plantar fasciitis | PRP FAST SYSTEM (BIO)/1 centrif. [Pl] ›10^6^/μl in 5 ml | 1Inj PRP + 2%xylocaine (=30) | At all time points PRP group had signif. lower VAS | --- | Short follow up, little inf. on interventions Poor PRP caracterization Scarcity of outcome measures No blinding | (+) |
| Antuña et al. 2013 [30] | RCT pilot (I)/n = 28 | 2 years | No PRP (n = 14) | Clinical ex., Arthro - MRI at 1 year, Constant and DASH | Rotator cuff massive tear | VIVOSTAT ®^2^ | 6.0 ml PRF at repair site (n = 14) | Signif. early improvements in Constant and VAS scores not sustained at 2 years follw up. Both groups wih ↑retear rate. | --- | Small N Little inf. on study design and PRP composition No blinding Poor PRP characterization | (−) |
| Magnussen et al. 2013 [31] | RETROSPECTIVE COMPARATIVE STUDY (III)/n = 100 | 2 years | No PRP(n = 50) | Clinical ex., KOOS, MARX, IKDC scores and KT-1000 | ACL rupture | GPS® II Platelet Concentrate Separation kit / Biomet^1^/ 1 centrif. | Reconstruction + PRP (n = 50) | Early improvement in joint effusion (10 ± 4D) not sustained at final follow up . Other outcome measures not affected by treatment. | --- | Not randomized, not blinded 42 patients lost at final folow up Some outcomes evaluated restropectively Little information on intervention and PRP composition | (−) |
| Mishra et al. 2014 [32] | RCT (I) /n = 230 | 24 weeks | Needling + 2–3.0 ml Saline Solution (n = 114) | >25% reduction of VAS (primarily), PRTEE and extended wrist examination | Chronic lateral epicondylar tendinopathy | Recover GPS, Biomet 3/ 1 centrif. / buffered with 8,4% sodium bicarbonate/ Le rich [Pl] ↑8X | Needling = 2–3.0 ml PRP inj. | Significant reduction in elbow tenderness in PRP group at 4, 12 and 24 weeksVAS signif.↓ at 8 and 24 weeks PRTEE not affected by treatment. At 24 weeks PRP group had higher success rate (P = .012) | OA*/P/TP and sponsor | Short follow up Poor PRP characterization Positive effect of needling alone Only clinical outcomes | (+) |
| Krogh et al. 2013 [33] | RCT (I) / n = 60 | 3 months | Saline 3.0 ml (n = 20) | PRTEE, US (thickness and color doppler) | Lateral epicondylitis | Recover GPS II, Biomet^1^ / 1 centrif. buffered with 8,4% sodium bicarbonate [Pl] ↑8X | I) 1 Inj. 3.0 ml PRP, buffered with NaHCO_3_ (n = 20) II) 1 inj. Glucocorticoid = 1 ml TA(40 mg/ml) + 2.0 ml lidocaine (n = 20) | At 3 months no difference between groups. Glucocorticoid group: short term pain ↓ (1 month), ↓ doppler activity, ↓tendon thickness | OA*/P | Short follow up due to signif. drop outs at 3 months Poor PRP characterization Consider effect of peppering tech. in saline and PRP groups | (−) |
| Patel et al. 2013 [34] | RCT (I)/ n = 78, 156 knees | 6 months | 8.0 ml Saline Solution Injection(n = 46) | WOMAC, VAS | KOA | 1 centrif/ WBC filtered/ [Le] = 0/ [Pl] ↑ 10x / MPC = 310,14 x 10^3^ mL/ CaCl_2_ | 1 inj. 8.0 ml PRP (n = 52) 2 inj. 8 ml PRP, 3 weeks apart (n = 50) | No differences between 1or 2 inj. of PRP Signif. early improvements deteriorate after 6 months PRP results better than saline | OA*/P | Only clinical outcome measures Short term follw up No randomization of knees | (+) |
| Wasterlain et al. 2012 [35] | RCT (I) / n = 11 | 12 weeks | Dry needling alone (n = 5) | VISA, TEGNER, LYSHOLM, SF-12 questionnaires and VAS. hGH, IGF-1 IGFBP-3, bFGF, VEGF, PDGF-BB in PRP and serum | Patellar tendinopathy | GPSIII System, Biomet^4^/ 1 centrif. /Le rich /GFs | PRP + dry needling under US guidance (n = 6) | VISA and TEGNER scores improve signif. in PRP + dry needling group | --- | Short follow up Small N Poor PRP characterization | (+) |
| Jain et al. 2012 [36] | RCT (I) /n = 75, 150 knees | 6 months | 8.0 ml Saline Solution Injection (n = 46 knees) | WOMAC | Early bilateral KOA | 1 centrif./ Le filtered/ CaCl_2_ | I) 1 inj. 8 ml PRP (n = 54 knees) II) inj. 8 ml PRP, 2–3 weeks appart (n = 25 knees) | PRP is more effective than placebo in pain, stiffness and mobility improvement in KOA patients Peak effect around 2–3 weeks with worsenig at final follow up | OA*/P | Short follow up Only subjective outcome measures Poor PRP characterization | (+) |
| Mardones et al. 2012 [37] | RTC (I) /n = 60 | 14 days | Saline Solution Injection (n = 20) | VAS / morfine requirements / Ecchymosis | Femoro acetabular Impingement | Platelet concentration measured | I) PRP clot (n = 20) II) PRP spray (n = 20) | Lower ecchymosis in PRP clot group No difference between treatments in other outcome measures | --- | Small N Very short follow up Scarcity of outcome measures No blinding Poor PRP characterization | (−) |
| Cerza et al. 2012 [38] | RCT (I-II) / n = 120 | 6 months | HA (20 mg/2 ml) IA 4 inj. weekly (n = 60) | WOMAC | Gonarthosis | ARTHREX®5/ 1 centrif. | 4 inj. of 5.5 ml PRP weekly (ACP) (n = 60) | PRP group had signif. effect after final inj. sustained up to 24 weeks, with better clinical outcomes (lower WOMAC scores) independent of gonarthosis level. | --- | Short follow up Poor hemoderivate caracterization Only subjective outcome measures No blinding No placebo control | (+) |
| Mei-dan et al. 2012 [39] | RTC (II) / n = 29, 30 OCLs | 28 weeks | HA (20 mg/2.0 ml) IA 3inj. weekly(n = 16) | AOFAS, AHFS, VAS and Subjective global function score | Talus osteochondral lesion | PRGF SYSTEM II, BTI^6^ / 1 centrif./[PL] 2-3X ↑ / CaCl_2_ | 3inj. 2.0 ml PRPIA, at 2 weeks intervals (n = 15) | VAS, AHFS improved signif. in PRP group as well as global function scores | --- | Short follow up Only subjective outcomes No blinding No placebo control | (+) |
| Almeida et al. 2012 [40] | RTC(I) / n = 27 | 6 months | No PRP (n = 15) | MRI, isokinectictesting, VAS, questionnaires | Patellar tendinopathy | Haemonetics MCS + 9000 cell separator^7^ + Platelet apheresis / MPC = 1.185.166/mm^3^ ± 404,472/mm^3^ (↑7,65)/ WBC = 0,91/mm^3^ ± 0,81 mm^3^ / CaCl_2_ + thrombin | 20 -40 ml PRP gel (n = 12) | Smaller gap area and lower VAS, post op, in PRP group No difference at 6 months between groups in questionnaires and isokinectic tests. No positive effect of PRP in patients’ early recovery | --- | Short follow up Small N No blinding | (±) |
| Spaková et al. 2012 [41] | CONTROLLED PROSPECTIVE COHORT(III)/ n = 120 | 6 months | 3 injections HA, weekly interval (n = 60) | WOMAC and NRS at 0, 3 and 6 months | KOA grade 1, 2, 3 (Kelgren and Lawrence classification) | 3 centrif./ MPC 680 ± 132X 10^6^ ml, [Pl] = ↑ 450%, WBC ↑ 3,6X | 3 PRP injections (n = 60) | PRP group had better WOMAC and NRS scores at 3 and 6 months follow up | --- | Poor study design No placebo control Short follow up No blinding Only subjective outcome measures | (+) |
| Aksahin et al. 2012 [42] | CONTROLLED PROSPECTIVE COHORT (III)/ n = 60 | 6 months | 2 ml of MPA (40 mg) + 2.0 ml Prilocaine (n = 30) | Roles and Maudsley scores, VAS 0, 3 weeks and 6 months | Plantar fasciitis | 2 centrif/ Calcium | 3.0 ml PRP + 2.0 ml Prilocaine (n = 30) | Both treatments reduce score signif but with no diferences between groups for outcomes mesuares | P/OA* | No placebo control Short follow up Only subjective outcome measures Poor PRP characterization | (−) |
| Rodeo et al. 2012 [43] | RCT (II)/ n = 79 | 12 months | Surgical repair only (n = 39) | US + doppler, ASES and L’Insalata Scores and strength measurements | Rotator cuff tear | CascadeAutologousPlatelet System^8^/ 2 centrif. / CaCl_2_ | PRFM atsurgicalrepair site (n = 40) | No signif. effect of PRFM demonstrated in outcome measures. Negative effect of PRFM suggested by analysis regression | P/OA* | Small N Poor PRFM characterization Premature US evaluation? | (−) |
| Weber et al. 2012 [44] | RCT (I) / n = 60 | 12 months | Repair without PRFM (n = 30) | ROM, SST, UCLA, SS, ASES scores (pre and postop) and MRI | Rotator cuff repair failure | CascadeAutologousPlatelet System^8^ /2 centrif. / ↓ Le/ CaCl_2_ | Application of commercially PRFM (n = 30) | No differences observed with PRFM application | P/OA* | Poor PRFM characterization | (−) |
| Bergeson et al. 2012 [45] | COHORT STUDY (III)/  n = 37 | 12 months | Repair without PRFM (n = 21) retrospectively selected | Functional outcome scores (UCLA, WORC, SANE, ASES, Constant) and MRI | Rotator cuff repair failure | CascadeAutologousPlatelet System^8^/2 centrif. / ↓Le/ CaCl_2_ | Application of PRFM to surgical site (n = 16) prospectively selected | PRFM did not result in improved retear rates or better funtional outcomes Delayed healing secondary to fibrin clot 10% infection rate in PRP treated patients | --- | Small N, no randomization, no blinding Poor PRFM characterization No placebo control Heterogeneity of repair techniques Side effects | (−) |
| Cervellin et al. 2012 [46] | RCT (I)/n = 40 athletes | 12 months | Reconstruction without PRP (n = 20) | VISA and VAS scores and MRI | ACL rupture and reconstruction | GPS® II System^1^/ 1 centrif./CaCl_2_ and thrombin | PRP applied after ACL reconstruction (n = 20) | ↓ pain (↑ VISA scores) in PRP group No difference in VAS scores between groups No signif. difference in bone defect filling | P/OA* for MRI | Small N Poor PRP characterization No placebo control | (±) |
| Filardo et al. 2012 [47] | RCT (I)/ n = 109 | 12 months | HA, 3injections, weekly (n = 55) | IKDC, EQ-VAS, TEGNER, KOOS scores, ROM and knee circunference at 0,2,6, and 12 months | KOA | 2centrif./ [Pl]↑ 5X, [Le] ↑ 1,2X | PRP, 3injweekly, (n = 54) | PRP results in improvement, not different from HA | P/OA* | Poor PRP characterization No placebo control | (−) |
| Jo et al. 2011 [48] | PROSPECTIVE COHORT CONTROL STUDY (II) / n = 42 | 16 months | Surgical repair without PRP (n = 23) | Pain, strength, ROM,ASES, UCLA, DASH, SST, SPADI, Constant, VAS and MRI | Rotator cuff tear | Plateletpheresis(COBE Spectra Turbo^9^)/ MPC = 1595,74 ± 454,08 / [Pl] 6,1x↑, Le = 0,38 ± 1,15 / Calcium gluconate 10% | Repair surgery with PRP [PL] = 1000 X 10^3^/μl (n = 19) | No statistical difference in outcomes between groups although MRI revealed lower retear rate with PRP treatment | --- | No randomization No blinding No placebo control | (−) |
| Randelli et al. 2011 [49] | RCT (I) / n = 53 | 24 months | Surgical repair without PRP (n = 27) | Constant, VISA, UCLA, VAS, SST, SER, MRI, US, clinical evaluation and retear rate at 0, 3, 6, 12 and 24 months. | Rotator cuff tear | GPS® II Biomet^1^ / 2 centrif./ CaCl_2_ | Surgical repair with PRP application (n = 26) | ↓ early post op pain (up to 30 days) but no difference at last follow up ↓Retear rate, but not signif. in PRP group Better outcome mesures in stage 1–2 cuff tears At 3 months only PRP group had signif. better clinical outcomes | OA*/P | Poor PRP characterization No placebo control | (+) |
| Castricini et al. 2011 [50] | RTC (I) / n = 88 | 16 months | Surgical repair without PRP (n = 45) | Constant score and MRI | Rotator cuff tear | CascadeAutologousPlatelet System^8^/ 2 centrif./ CaCl_2_ | Intraoperative application of PRFM (n = 43) | No signif. difference in outcome measures between groups | OA* | Poor PRP characterization No placebo control | (−) |
| Thanasas et al. 2011 [51] | RCT (I) / n = 28 | 6 months | 3.0 ml ABI inj., US guided (n = 14) | VAS, Liverpool elbow score | Chonic lateral elbow epicondylitis | GPS® III Biomet^1^/ 1 centrif. / [Pl]1.292.500/ml  MPC ↑5,5 x / [Pl]/[Le] = 111/1 / GFs: EGF 3,9x↑, IGF-1 1x↑, VEGF 6,2x↑, TGFβ1 3,6x↑, PDGF not applicable | 3.0 ml PRP with US guided (n = 14) | VAS better in PRP group, but signif. only at 6 weeks | OA* | Small N Only subjective outcome measures No placebo control Short follow-up | (−) |
| Schepull et al. 2011 [52] | RCT (II)/ n = 30 | 12 months | Surgical repair (n = 14) | Elasticity modulus (CT), RSA, ATRS, Heel Raise Index | Achilles tendon rupture | 2centrif./ MPC =3673 ± 1051 x 10^9^/ mL / CaCl_2_ | Surgicalrepair + 6.0 ml PRP (n = 16) | No difference in elastic modulus or heel raise index PRP had a detrimental effect on ATRS | OA*/P | Small N No placebo control 4 patients lost in PRP group at 52 weeks | (−) |
| de Vos et al. 2011 [53] | RCT (I) / n = 54 | 24 weeks | Saline injection + excentric exercise (n = 27) | VISA-A, UTC and Colour doppler | ChronicAchilles tendinopaty (midportion) | Recover Platelet Separation Kit^11^/ 1centrif. | 4.0 ml PRP inj. US guided + eccentric exercise (n = 27) | No signif. difference between groups | P/OA*/TP | Poor PRP characterization Short follow up | (−) |
| Creaney et al. 2011 [54] | RTC (I) / n = 150 | 6 months | 2 ABI inj. at 0 and 1 month + 2 ml bupivacaine, US guided (n = 70) | PRTEE at 0, 1, 3 and6months | Elbow tendinopathy | 1centrif. / MPC : PRP 652 x 10^9^/l (2,8x↑) / MPC: ABI 234 x 10^9^/l | 2 PRP inj. at 0 and 1 month + 2.0 ml bupivacaine, US guided (n = 80) | No signif. difference between treatments Both improved PRTEE | OA* | Incomplete PRP characterization Short follow up No placebo control Scarcity of oucome measures | (−) |
| Kon et al. 2011 [55] | PROSPECTIVE COMPARATIVE STUDY (II) / n = 150 | 6 months | HA HMW (n = 50) HA LHM (n = 50) | IKDC and EQVAS scores and adverse events; overall satisfaction | KOA (early and severe) | 2centrif. / [Pl] ↑600% / CaCl_2_ | 3 PRP inj every 14 days, average 6 x 10^9^ platelets injected per treatment (n =50) | PRP showed more and longer efficacy than HA in reducing pain, symptons and restoring function Better results in younger, active and less affected patients | --- | No placebo control Short follow up Poor PRP characterization No blinding No randomization Only clinical outcomes | (+) |
| Jonge et al. 2011 [56] | RCT (I) n = 54 | 12 months | 4.0 ml Saline inj. + marcaine, US guided (n = 27) | VISA-A, UTC and patient satisfaction | Achilles tendon insertion tendinopathy | Recover Platelet Separation Kit^11^ / 1 centrif./ NaHCO_3_ buffer | 4.0 ml PRP + NaHCO_3_ + marcaine, US guided (n = 27) | No clinical or sonografic benefit from PRP treatment found | P/OA*/TP | Poor PRP characterization Small N | (−) |
| Gosens et al. 2011 [57] | RCT (I) / n = 100 | 24 months | 1.0 ml TA (40 mg/ml) + bupivacaine, peppering needling technique (n = 49) | VAS and DASH 25% reduction and 2 years without reintervention | Chronic lateral epicondylitis | RecoverPlatelet System^11^/ 1 centrif. | 5.0 ml PRP inj. + 3.0 ml buffered NaHCO_3_ 8,4% + bupivacaine HCl (n = 51) | Corticosteroid group better initially and then declined, while PRP progressively improved | P/OA* | No placebo control Poor PRP characterization Only clinical evaluation | (+) |
| Barber et al. 2011 [58] | CASE CONTROL STUDY (III) / n = 40 | 24 - 44 months | Reconstruction without PRFM (n = 20) | ASES, SANE, ROWE, SST, Constant scores and MRI | Rotator cuff tear | CascadeAutologousPlatelet System^8^ / 2 centrif. / CaCl_2_ | Repair with 2 PRFM constructs (n = 20) | No signif. difference in clinical outcome scores between groups PRFM treatment ↓ incidence of MRI - verified retears. | --- | Poor PRP characterization No blinding No randomization Small N | (+) |
| Horstmann et al. 2011 [59] | RCT (I) / n = 40 | 15 days | No PRP gel after arthropasty (n = 20) | Postop Hb loss and pain,transfusions, VAS, ROM, hospital stay, hematomas, healing disturbances | KOA (total kneearthroplasty) | GPS® system^1^/ 2 centrif./ CaCl_2_ and thrombin | Platelet gel applied after artroplasty (n = 20) | Significant improvement not found . PRP ↓VAS and haematomas signif.on day 3postop. only | P/OA* | Poor PRP characterization Small N Short follow up | (−) |
| Buford 2011 [60] | PROSPECTIVE COHORT CONTROL STUDY (II) / n = 100 | 6 months | Surgical repair n = 50 | US Healing time | Rotator cuff tear up to 3 cm in length | --- | Surgical repair + 5–6.0 ml of PRP (n = 50) | PRP had no significant effect on outcome measures | --- | No PRP characterization No blinding No placebo control | (−) |
| Vogrin et al. 2010 [61] | RCT (I) / n = 50 | 12 weeks | No Platelet gel (n = 25) | Contrast -enhanced MRI (graft revascularization) | ACL rupture | Magellan Autologous Platelet Separation^12^ / MPC = 978 x 10^9^/l/  Thrombin | 6.0 ml Platelet gel applied into femoral and tibial bone tunnels (n = 25) | Higher vascularization in PRP group at 4-6w, postop in the osteoligamentous interface. PRP did not favor revascularization of intraarticular part of graft. | P/OA* | Small N No histology Only one outcome measure Short follow up | (±) |
| Peerbooms et al. 2010 [62] | RCT (I) / n = 100 | 12 months | TA (40 mg/ml)  + Bupivacaine and epinephrine with peppering technique (n = 49) | 25% VAS and DASH reduction without a reintervention after 1 year | Chronic lateral epicondylitis | Recover Platelet Separation kit^11^/ 1centrif. | PRP + NaHCo_3_ 8,4% + bupivacaine and epinephrine with peppering technique (n = 51) | Corticosteroid group was better initially and then declined. After 26 PRP group had signif.better VAS and DASH scores and sustained results until end of study. | P/OA* | No placebo control Only clinical outcomes Poor PRP characterization | (+) |
| de Vos et al. 2010 [63] | RCT (I) / n = 54 | 24 weeks | 4.0 ml Saline, US guided inj + marcain + eccentric exercise (n = 27) | VISA - A Patient satisfaction and return to sports activity | Chronic midportion Achilles tendinopathy | Recover Platelet separation kit^11^/ 1 centrif. / Microbial culture | 4.0 ml PRP + 8,4% NaHCO_3_ US guided inj + marcain + excentric exercise (n = 27) | Improvement not signif. different between groups for all outcome measures | P/OA*/TP | Subjective outcome measures Poor PRP characterization Short follow up No placebo group Effect of excentric exercise | (−) |
| Radice et al. 2010 [64] | PROSPECTIVE CASE CONTROL STUDY (III) / n = 50 | 12 months | No PRP gel added to graft (n = 25) | MRI | ACL rupture | GPS System (Biomet^1^), 1 centrif/ Activating agent not reported | 5.0 ml PRP added to graft (n = 25) | Statistically shortened time for graft maturation with PRP gel use (48% reduction) | OA* | Small N Poor PRP characterization No placebo control Scarcity of outcome measures Poor study design | (+) |
| Filardo et al. 2010 [65] | PROSPECTIVE CONTROLLED STUDY (II) / n = 31 | 6 months | Physiotherapy only(n = 16) | EQ - VAS and Tegner scores, functional recovery and patient satisfaction | Patellar tendinopathy | 2centrif/ [PL]) 6,1 ± 1,6 X↑ CaCl_2_ | 3.0 ml PRP - 3 inj. every 15 days (Average of 6,5 ± 1,5 X 10^6^ platelets administered per treatment) + physiotherapy (n = 15) | Patients in both groups showed improvement in outcome measures without signif. difference, except for sport activity level, better in PRP group | --- | Small N No blinding Short follow up No placebo control | (−) |
| Nin et al. 2009 [66] | RCT (I) / n = 100 | 24 months | ACL reconstruction without PRP gel (n = 50) | Clinical evaluation (pain, laxity, IKDC) C-PR, Knee perimeter, KT-1000, radiology evaluation, MRI | ACL tear | 2centrif. /MPC = 837 X 10^3^/ mm^3^/ [Pl] 469% ↑/ CaCl2082 | ACL reconstruction with PRP gel (n = 50) | No significant difference in clinical or biochemical outcomes between groups. | P/OA* | No placebo control | (−) |
| Silva; Sampaio 2009 [67] | RTC (I) / n = 40 | 3 months | Anatomic ACL reconstruction without PRP (n = 10) | MRI at 3 months | ACL tear | Mini GPS®III kit (Biomet®^1^) 1 centrif. / Thrombin | I) 3.0 ml PRP at the end of surger(n = 10)II) 3.0 ml PRP at end of surgery and after 2 and 4 weeks (n = 10)III) 3.0 ml PRP activated with trombin at end of surgery (n = 10) | No significant difference between groups in MRI | --- | Small N Short follow up, no blinding Poor PRP characterization Scarcity of outcome measures | (−) |
| Peerbooms et al. 2009 [68] | RCT / n = 102 | 3 months | No PR gel (n = 52) | VAS, WOMAC and postop. wound scores, knee funcion, Hb levels | Total knee arthroplasty | GPS® System Biomet^1^ / 1 centrif./ CaCl_2_ and thrombin | 6.0 ml PR gel applied to the surgical site (n = 50) | No significant differences between groups No PR gel effect on outcome measures | P/OA* | 18 drop outs in PG group Short follow up Poor PRP characterization | (−) |
| Sanchez et al. 2008 [69] | OBSERVATIONAL RETROSPECTIVE COHORT / n = 60 | 1,5 months | 3 inj. HA, weekly (n = 30) | WOMAC questionaire at 0 and 5 weeks; 40% reduction from baseline in joint pain (WOMAC subscale), joint stiffness, physical function and global WOMAC | KOA | 1centrif./ CaCl_2_ / [Pl] ↑2X/ Le = 0 / MeanGFs: PDGFAB 17,41 ± 9,66 ng/cc, TGFβ1, 29,15 ± 12,88 ng/cc, VEGF 212 pg/cc. IGF-I, HGF = plasma levels [Pl]↑ 2 ± 0,5 X, No WBC | 6-8.0 ml PRGF inj. weekly, for 3 weeks (n = 30) | PRP group had better WOMAC score, especially in pain and physical function subscales | --- | No placebo control No randomization No blinding Only clinical and retrospective evaluation Poor study design Short follow up | (+) |
| Everts et al. 2008 [70] | RCT (II) / n = 40 | 3 months | Open subacromial decompression without PRP (n = 20) | ASES, joint instability, pain, pain medication and ROM | Chonic impingiment syndrome | Magellan Autologous Platelet Separator System^12^ / 1 centrif./ MPC = 1.183 ± 396 x 10^9^/l, [Pl]↑5,7x / [Le] ↑2,7x/ Thrombin | Open subacromial decompression + PRP (n = 20) | Platelet-leukocyte gel group had signif. improvement in ASES score, pain reduction, faster ROM recovery and normalization of ADL | P | Small N Only clinical evaluation Short follow up | (+) |
| Orrego et al. 2008 [71] | RCT (II) / n = 108 | 6 months | ACL reconstruction (n = 27) | MRI at 3 and 6 months, IKDC and Lysholm scores | ACL tear | Biomet GPSII®^1^ kit/ 2 centrif. /Thrombin and CaCl_2_ | I) PC + ACL reconstruction (n = 26) II) BP + ACL reconstruction (n = 28) III) PC + BP + ACL reconstruction (n = 27) | No difference between groups at 3 and 6 months. PC had a signif. enhancing effect on graft maturation when compared to control | OA* | Short follow up Incomplete blinding No histological or biomechanical evaluation Poor PRP characterization | (−) |
| Sanchez et al. 2007 [72] | RETROSPECTIVE CASE CONTROL STUDY (III) and descriptive laboratory study / n = 12 athletes | 12 months | Conventional suture repair n = 6) | ROM, functional recovery, US and complications | Achilles tendon tear | PRGF SYSTEM II^6^/ 1 centrif./ MPC = 634 x 10^3^ ± 217 x 10^3^/μl,[Pl] ↑3,1x, no WBC / GFs: IGF-1 94,53 ± 32,84 ng/mL, TGFβ1 74,99 ± 27,48 ng/ ml, PDGFAB 35,62 ± 14,57 ng/mL, VEGF 383 ± 374 pg/mL, HGF 593,87 ± 155,8 pg/mL, EGF 481,5 ± 187,5 pg/mL/ CaCl_2_ | Open suture repair + PRGF (n = 6) | PRGF treated athletes regained ROM signif. earlier and returned ealier to running and training activities and tendons had ↓ CSA. GFs concentrations in PRGF were significantly correlated with number of platelets | --- | No randomization No blinding Small N, retrospectively evaluated No placebo control Poor study design | (+) |
| Everts et al. 2007 [73] | OBSERVATIONAL CONTROLLED STUDY (III) / n = 165 | 5 months | No platelet gel after surgery (n = 80) | Post op. blood loss (Hb), ROM, incidence of arthrofibrosis | Total knee arthroplasty | Electa ^TM13^, 1 centrif./ CaCl_2_/Thrombin | Platelet gel at end of surgery (n = 85) | Significant less ↓ Hb loss and arthrofibrosis, signif.↑ ROM and shortened hospital stay in PRP gel group | --- | No randomization No blinding No placebo control Poor PRP characterization Short follow up | (+) |
| Gardner et al. 2007 [74] | RETROSPECTIVE CASE CONTROL STUDY (III) / n = 98 | 15 days | No platelet gel after surgery (n = 37) | Blood loss (Hb), ROM, narcotic use, arthrofibrosis, hospital stay | Total knee arthroplasty | Medtronic Sequestra 100, Autotransfusion System^14^/ MPC = 785.500/ ml/ [Pl] 338% ↑ /Thrombin | 8.0 ml Platelet gel at end of surgery (n = 61) | ↑ROM,↓in blood loss,↓ pain medication and ↓lenght of hospital stay in PRP gel treated patients | --- | No randomization No blinding Short follow up Indirect pain measurement Small control group No placebo control Poor study design | (+) |
| Zavadil et al. 2007 [75] | RCT (II) / n = 40 | ‹ 15 days | No treatment after surgery (n = 20) | Blood loss, ROM, VAS, length of hospital stay | Total shoulder arthroplasty | Magellan Autologous Platelet Separation System^12^ / 1 centrif./ [Pl] 4,26 X↑, [Le] 2,41 X↑ in PRP / Thrombin/ CaCl_2_/ MPC = 1090,94 ± 388,19 and [Le] 16,80 ± 11,75 in PRP/ MPC = 54,97 ± 28,11 and [Le] 0,32 ± 0,37 in PPP | Autologous platelet gel and PP after surgery (n = 20) | Significant ↓ in VAS and narcotic use. ↑ROM in Platelet gel and PP group | P/OA* | Small N Only clinical evaluation Short follow up | (+) |
| Mishra; Pavelko 2006 [76] | PROSPECTIVE COHORT STUDY (III) n = 20 | 24 months | 2-3.0 ml local anestheticinjection (bupivacaine + epinephrine) (n = 5) | VAS, MayoElbowscore | ChronicElbow tendinopathy | GPS® System^1^/ 1 centrif./ [Pl] 539%↑ | 2-3.0 ml PRP injection (average 3.31 million platelets/ treatment) (NaHCO_3_ buffered) (n = 15) | PRP group had 60% improvement in pain scores at 8 weeks, 81% at 6 months and 93% at longer than 1 year, and signif. improvement in fuction scores. | --- | Small N Expressive control drop outs (60%) No randomization No blinding Poor PRP characterization Only clinical outcomes Poor study design | (+) |
| Ventura et al. 2005 [77] | RCT (II) / n = 20 | 6 months | No GF treatment (n = 10) | KOOS, KT-1000, Tegner, CT, clinical exam, MRI occasionally | ACL tear and laxity | GPS®Biomet System^1^/ 1 centrif. | GF-treated ligaments (n = 10) | No diferrence in funcional or clinical evaluation; ↑ ACL density on CT in GF-treated group. | --- | Small N Short follow up Poor PRP characterization No blinding No placebo control | (−) |

[]:concentration; ↑:increase; ↓: decrease.

^1^ Biomet Corporate Headquarters, Warsaw, Indiana, USA; ^2^Vivostat, Copenhagen, Denmark; ^3^Biomet Corporate Headquarters; ^4^Biomet Corporate Headquarters; ^5^Arthrex, Naples, Florida, USA; ^6^Endoret BTI Biotechnology, San Antonio, Spain; ^7^Haemonetics Corporation, Braintree, MA, USA; ^8^MTF Sports Medicine Edison, New Jersey, USA; ^9^Terumo BCT, Mississauga, Ontario, USA; ^10^Pall, Port Washington, New York, USA; ^11^Blood Recovery Systems, Fort Myers, Florida, USA; ^12^ Magellan, Hopkinton, MA, USA; ^13^ Sorin Group, Mirandola, Italy; ^14^Medtronic Parkway, Minneapolis, Minnesota, USA.
