# Supplementary material for: Platelet-rich plasma in orthopedic therapy: a comparative systematic review of clinical and experimental data in equine and human musculoskeletal lesions
Source: BMC Vet Res. 2015 Apr 22;11:98. doi: 10.1186/s12917-015-0403-z (PMC4449579; doi:10.1186/s12917-015-0403-z)
Supplement: Additional file 2: Table S2. — Characteristics of 63 experimental studies that provided evidence regarding PRP intervention. [file 12917_2015_403_MOESM2_ESM.doc]

| Authors/ year | Study design | Population/ tissue | Control | Outcome measures | Hemoderivate acquisition/ cytology/ analysis/composition | Intervention | results | prp effect |
| --- | --- | --- | --- | --- | --- | --- | --- | --- |
| **Textor; Willits; Tablin 2013** [78] | Controlled*in vitro* and *in vivo* 5 days | Horses (n = 7)  28 healthy metacarpo/ metatarsophalangeal joints for *in vivo* exp.(I) and 14 intercarpal joints for *in vitro* exp.(II) | I)Saline (n = 7 joints)  II)1 ml Saline + 1.5 ml SF | I) IL-6,TNF-α, PDGF-BB and TGFβ1 in SF at 0,6,24,48 and 96 hs post injection  II) PDGF, TGFβ, IL-6, TNF-αand IL-1β | E-PET™^1^ / MPC = 650 ± 242,6 X 10^3^/ μL, Le =14,8 ± 3,84 X 10^3^μL / PDGF, TGFβ/ IL-6, TNFα and IL-1β | IA) Resting PRP (n = 7 joints)  IB) CaCl₂ activated PRP(n = 7 joints)  IC) Thrombin activated PRP (n = 7 joints) (2 ml injs.)  IIA) Resting PRP  IIB) SF+ PRP | I) PDGF was not increased with any treatment and TGFβ increased at 6 h in resting PRP and Thrombin activated PRP and at 24 h in resting PRP, but at 96 h GFs were not different in PRP treated and control joints.  Thrombin activation ↑TNFα at 6 h and IL-6  PRP injection resulted in transient joint effusion with ↑ TNFα and IL-6 at 6 h  II) [PDGF] and [TGFβ] in PRP + SF were signif.↑ than controls and baseline No difference in inflammatory cytokines | (±) |
| **Textor; Tablin 2013** [79] | Controlled *in vivo* 5 days | Horses (n = 7) 28 healthy metacarpophalangeal joints | Saline (n = 7 joints) | Clinical examination and laboratorial analysis (blood + SF) | E-PET™^1^ / MPC = 542 ± 196,3 X 10^3^/ μL, [Pl] ↑ 3,2X, Le 13,1 ± 3,46 X 10^3^μ, [Le]↑1,9X / PDGF, TGFβ | I) Resting PRP (n = 7 joints)  II) CaCl₂ activated PRP (n = 7 joints)  III) Thrombin activated PRP (n = 7 joints) (2.0 ml injs.) | GFs signif. ↑over control levels in all activated treatments, more so with thrombin activation HR ↑ RR ↑ at 6 h after PRP treatments  Thrombin activation signif. associated with ↑effusion,↑TP, pain and periarticular swelling (other groups had effusions only at 6 h) WBC ↑ at 6 and 24 h in all groups, greater in thrombin activted PRP, lasting for 48 and 96 h in activated PRPs  [Pl] ↑ after injection | (±) |
| **Zandim et al. 2013** [80] | Controlled *in vivo* | Horses (n = 6)  Experimentally induced SDFT tendinopathy | 1.8 ml Saline injected into lesions (n = 6) | Physical examination, lameness evaluation and US.  Histologic evaluation at 3 and 16 days after treatment (HE, Massom’s trichome and Picrosirius Red), and immunohistochemical examination | 2centrif. / MPC = 368 .333 ± 39.707/μL | 1.8 ml PRP inj. into lesions 5 days after lesion induction (n = 6) | Histological, morphometric and immunihistochemical results were not influenced by a single PRP injection | (−) |
| **McCarrel et al. 2012** [81] | Controlled *in vitro* | Horses (n = 7)  SDFTs | Tendon cells in culture media | COL1A1, COL3A1, COMP, MMP-13, IL-1β and TNF-α mRNA expression | SMART PREP®^4^ 2 system / 1 centrif./ Plaq /Le/ PDGF- BB | Culture media with fresh and 100%:  I) Intermediate concentration PRP  II) Le reduced PRP III) Le concentrated PRP  IV) ↑[Pl] ↑[Le] PRP (same PL: Le as group 1) | COMP, COL1A1: COL3A1 and MMP-13 expression in culture media did not differ between PRP groups  COMP, COL1A1:COL 31A1 were ↑ in all PRP groups compared with control  COL1A1 expression was signif.↑ in Le reduced PRP  MMP-13 was ↓ in PRP groups compared to control  IL-1β was lowest in Le reduced PRP and highest in Le concentr PRP  TNF-α was lowest in Le reduced and control groups | (+) |
| **Yamada et al. 2012** [82] | Controlled *in vivo* | Horses (n = 4)  Osteochondral defects in lateral femoral throclea | Saline treatedjoints (n = 2) | Clinical and synovial fluid evaluation.  Macroscopic, histologic and histochemical evaluation at 0 and 150 days | 2 centrif/ MPC = 362.350/μl / CaCL₂ and thrombin at day 30 only | Intralesional (day 30) and intra articular PRP at days 30, 45, 60 and 75 (n = 2) | Improved lameness scores, macroscopic, histological and histochemical features in PRP treated group  Better tissue repair in PRP treated group Treatment had no significant results on synovial inflammation | (+) |
| **Bosch et al. 2012** [83] | Controlled *in vivo* 24 weeks | Horses (n = 6)  Surgically created SDFT lesions | Saline, 3.0 ml injection 1 week postop., US guided (n = 6) | US and US doppler at 2,3,5,8,12,18 and 24 weeks postop, immunohistochemical  (angiogenesis) and histological evaluation | GPS II, Biomet^8^/ 1 centrif. | 3.0 ml PRP inj. 1 week postop., US guided (n = 6) | PRP group ↑ blood flow except at week 5  PRP promoted neovascularization, higher number and better structural organization of blood vessels | (+) |
| **Bosch et al. 2011** [84] | Controlled *in vivo* 24 weeks | Horses (n = 6)  Surgically created SDFT lesions | Saline, 3.0 ml injection 1 week postop., US guided (n = 6) | UTC, Histology (HE) at 24 weeks | GPSII^8^/ 1 centrif./ PI] 3,8 X↑ | 3.0 ml PRP inj. 1 week postop., US guided (n = 6) | Better histological fiber arrangement in PRP group  PRP group had↑ fibrillogenesis and collagen formation  ↓Fluid and cellular accumulation in PRP group | (+) |
| **Maia et al. 2009** [85] | Controlled *in vivo* | Horses (n = 6)  Collagenase induced SDF tendinopathy | Saline injection (2.5 ml) 12 days after induction of lesion (n = 6) | Clinical, US and histologic evaluation 36 days after treatment | 2centrif. + 1 centrif. / MPC = 407,500 ± 58,800/μl / CaCl₂ | PRP inj.(2.5 ml), 12 days after lesion (n = 6) | PRP treated tendons had signif. better organization and paralelism of collagen fibers and fibroblasts at 36 days. | (+) |
| **Mc Carrel; Fortier 2009** [86] | Controlled*in vitro* | Horses (n = 5)  SDFT and SL explants | Explants cultured in 10% plasma in DMEM | Gene expression for CO1A1, COL3A1, COMP, Decorin, MMP3, MMP13 | Smart PReP 2 System®^4^ /1 centrif./ [Pl]↑5,54 X in PRP / | Culture media with:  I) PRP  II) 100% BMA  III) 100% PRP  IV) PPACD  V) PPCPD | Signif. ↑ gene expression of COMP and decorin and ↓MMP 3 expression with PPs and PRP support their beneficial effects in tendon and ligament healing.  PPs and PRP release the greatest concentrations of GFs.  PPs and PRP result in ↑ COL1 A1expression and ↓ COL3A1. | (+) |
| **Schnabel et al. 2008** [87] | Controlled *in vitro* | Horses Suspensory ligament explants | Culture in 10% plasma | PCR (COL1A1 and COL3A1, COMP, decorin, MMP3 and MMP13), total DNA and collagen. | Smart PReP2 System^4^/ 1 centrif./ TGF1, TGFβ1, PDGF-BB | Culture media with blood, PPP,PRP ABM at 10%, 50%, 100% and plasma at 50% and 100% | ABM at 100% stimulated decorin and COMP synthesis more than any other treatment, being preferred over other blood products for SL regeneration | (±) |
| **Schnabel et al. 2007** [88] | Controlled *in vitro* | Horses SDFT explants | Culture in 10% plasma | PCR (COL1, COL3, COMP, MMP3, MMP13, decorin) cell proliferation, total collagen | Smart PReP2 System^4^/1 centrif./ [Pl] ↑ 3,77 X/ MPC =395 x 10^3^/μl/ IGF-I, TGF-β1 ↑2X, PDGF-BB↑3,12X | Culture media with blood, PRP, PPP or BMA at 100%, 50% and 10% and plasma at 50% and 100% | Culture with PRP at 100% had increased COL1A1, COL3A1 and COMP with no increase in MMPs. PRP had signif. ↑ [TGFβ1 and PDGFBB] | (+) |
| **Smith; Ross; Smith 2006** [89] | Controlled *in vitro* | Horses,’ Suspensory ligament fibroblasts | Fibroblasts cultured in medium solution | COMP synthesis; ^3^H leucine incorporation | [Pl] PRP › ≥ 4X[Pl] blood | Fibroblasts cultured in 5 and 10% ABM, 5 and 10% PRP, 10% FS and 10% ES | PRP demostrated signif. anabolic effects on SL fibroblasts but was not as effective as ABM regarding COMP synthesis and ^3^H leucine incorporation | (±) |
| **Bosch et al. 2010** [90] | Controlled *in vivo* 24 weeks | Horses (n = 6)  Surgically created SDFT tendonitis | Placebo injection (3.0 ml, US guided), 1 week postop. (n = 6) | Lameness score, clinical, biomechanical, biochemical and histological evaluation. GAG, collagen, cross-links and DNA contents | GPS II Biomet^8^/ 1 centrif./ [Pl] ↑3,78x, [Le] ↑6x / PDGF-BB ↑ 2,94 X; TGF-β1 ↑4,47 X; IGF-1 ↑ 0,71X | 3.0 ml PRP inj., 1 week postop. (US guided) (n = 6) | PRP group had signif. ↑ GAG, collagen, cellularity and vascularity.  Biomechanical paramethers were improved (elastic modulus and stress at failure) with PRP treatment.  PRP Improvedhistologicalorganization. | (+) |
| **Sadoghi et al. 2013** [91] | Controlled*in vitro* 21 days | Human torn rotator cuff fibroblasts (n = 6) | --- | GAG and DNA, measurements at 1,7,14 and 21 days | 1centrifugation | Cell cultures supplemented with PRP concentrated 1,5 or 10X of the initial blood sample | ↑ Proliferation and activity of fibloblasts, with ↑ GAG and DNA levels at 1 or 5 fold of PRP concentration | (+) |
| **Muto et al. 2013** [92] | Controlled*in vitro* 21 days | Human rotator cuff derived cells from torn supraspinatus tendons (n = 4) | Cells cultured in regular medium | Cell morphology and viability, detection of apoptosis | 2 centrif. / [Pl] 510 -744% X↑, (736.000 - 1.449.000 pl/μl)/ Thrombin | I) 0.1 mg/ml TA in culture media  II) 0.1 mg/ml TA in culture media + 10% PRP  III) 10% PRP in culture media | TA decreases cell viability and increases apoptosis, both prevented by addition of PRP to culture media | (+) |
| **Carofino et al. 2012** [93] | Controlled*in vitro* | Human tenocytes from Biceps tendons (n = 4) | Tenocyte culture with:  I) Saline  II) Lidocaine 1%  III)Bupivacaine 0,5%  IV) MPD 40 mg/ml  V) Lidocaine + MPD  VI) Bupivacaine + MPD | Tenocyte proliferation (incorporation of radioactive thymidine;) cell viability (luminescence assay) | PRP single spin (SS) :(ARTHEX®^5^), 1 centrif./ MPC = 361,5 x10^3^/μl[Pl]↑2,6X; Le = 0,6^6^x10^3^/μl  PRP double spin (DS):MPC 447,7x10^3^/μl; [Pl] ↑3,3X; [Le], 0,17x 10^3^/μl | Tenocyte culture with PRP SS and PRP DS plus:  I) Lidocaine 1%  II) Bupivacaine 0.5%  III) MPD 40 mg/ml  IV) Lido + MPD  V) Bupi + MPD | Addition of anaesthetics and /or corticosteroids ↓ tenocyte proliferation and viability, previously stimulated by PRP | (+) |
| **Jo et al. 2012** [94] | Controlled *in vitro* | Human torn rotator cuff tendons (n = 9) | Tenocytes cultured in FBS | Tenocyte proliferation; total collagen and GAG synthesis. Gene expression in culture media at 7 and 14 days for COL I and COL III, decorin, tenascin C and scleraxis | COBE Spectra LRS Turbo^6^ / MPC = 1460 x 10^3^/μl [Pl]↑ 5,5 X in PRP group and [Pl] 1000 x 10^3^/μ in PPP group/ Ca gluconate/ Thrombin | Tenocytes cultured with 10% PPP, PRP+ Ca and PRP+ Ca + T at [Pl] of 100, 200, 400, 800, 1000, 2000, 4000, 8000 and 16000 x10^3^ Pl/μl, for cell proliferation assay.  Tenocytes cultured with PPP or PRP at [Pl] 1000Pl/μl + Ca or Ca + T for gene expression assay | Tenocyte proliferation, COLI at day 7, COLIII at day 7 and 14 ↑ in PRP treated cultures. Dose dependent response  The addition of thrombin increased cell proliferation  Decorin and scleraxis ↑ day 14  GAG ↑ at 14 days  Total COL synthesis ↑ 7–14 days | (+) |
| **Zhai et al. 2012** [95] | Controlled*in vitro* | Human semitendinous and gracilis tenocytes | Tenocytes/tenocytes and osteoblasts separated by filter, cultured without PRP | Cell proliferation and immunostaining (COL I, vimentin, COL III, VCAM) | 2centrif. MPC = 1005 x 10^12^/L | Culture media (Coculture):  I) Tenocytes + PRP  II)Osteoblasts + PRP | Proliferation rate was was lowest with 2 cells in coculture without PRP  PRP ↑proliferation rates of tenocytes and osteoblasts and ↓inhibition of cytokine mediated cell growth | (+) |
| **Wang et al. 2012** [96] | Controlled *in vitro* | Human harmstring tenocytes (n = 11) | Tenocytes cultured in 10% FBS | Cell proliferation, viability, collagen production, and Scleraxis, COL I, COL III and decorin mRNA expession | 2centrif. / plat 4x/ CaCl_2_ | Tenocytes cultured with 1%, 5% or 10% PRP | 10% PRP ↑ cellproliferation at day 7  10% PRP group had greatest collagen production and greatest expression of tenocyte markers |  |
|  | Controlled *in vivo* | Mice (n = 15)  Diffusion chambers(DC) containing tenocytes precultured in different media implanted into mice’s abdominal cavities | I) Mice impanted with DC containing tenocytes cultured with FBS (+ control) (n = 5)  II) Mice implanted with DC without tenocytes (−control) (n = 5) | Histological, immunohistochemichal and ultra-structural evaluation.  Scleraxis, COL I, COL III anddecorinmRNAexpession | 2centrif. / [Pl]↑ 4x/ CaCl2 | Mice implanted with DC containing tenocytes precultured with 10% PRP (n = 5) | PRP induced more collagen fibril formation  No signif. difference in gene expression compared with control | (±) |
| **Baboldashti et al. 2011** [97] | Controlled*in vitro* | Human harmstring tenocytes (n = 3) | No PRP in culture, only ciprofloxacin or dexamethasone | Tenocyte viability, senescence and cell death | 1 centrif./ [PI] = 353 x 10^3^/μl −837 x10^3^/μl / thrombin | Cell culture with:  I) PRP  II) ciprofloxacin + PRP  III) dexamethasone + PRP | PRP ↑ cell viability, even in the presence of ciprofloxacin and dexamethasone, in a dose dependent manner  PRP ↓ senescence when added to dexamethasone supplemented culture  PRP↓ cell death when added to ciprofloxacin supplemented culture | (+) |
| **Van Bull et al. 2011** [98] | Controlled *in vitro* | Human chondrocytes cultured with IL-1β | No PRP in culture media | COL 2 AI, aggrecan, ADAMTS 4 and 5, PTGS₂, GA and MMP-13 expression, NO production; NFKB activation | GPSIII System^9^/ 1 centrif./ [Pl]↑6,04 - 7,75x / citology/ CaCl₂ / VEGF, PDGF-AA,PDGF- AB/BB AND TGF-β1 | Culture medium supplemented with 1% or 10% PRP releasate (PRPr) | PRP has antiinflamatory proprieties (via inhibition of IL-1βinduced NFKB activation) and positevely affects gene expressionaffected by IL-1β of COL2A1 aggrecan, ADAMTS 4 and PTGS3 | (+) |
| **Wu et al. 2011** [99] | Controlled*in vitro* | Human chondrocytes cultured with IL-1βand TNF-α | Cell cultures without PRP or collagen matrix | Cell proliferation, and viability; COL 2, SOX9, COX2, IL-1β, aggrecan, MMP-2 gene expression and integrin β1α1 immunostochemistry | MCS blood cell separation system/ ^10^ thrombin/ TGFβ1 | Culture medium supplemented with PRP and collagen matrix | PRP group ↑ cell viability (dose dependent response).  PRP treatment reverted cell proliferation reduction incited by IL-1β and TNF-α, as well as reduced COL II and aggrecan expression.  PRP inhibited expression of COX2 and MMP-2  Collagen matrix enhance and modulate the regenerative potential of PRP. | (+) |
| **Bendinelli et al. 2010** [100] | Controlled*in vitro* | Human chondrocytes cultured with NF-κβ | Culture without PRP | Immunofluorescence, COX2, CXCR4 gene expression | GPSII Biomet^8^/ 1 centrif./ [Pl] 8x ↑/ Thrombin e CaCl₂; HGF, PDGF, TGF-β1, TNF-α, IL-1α, IL-1β, MCP-1 and EGF | Culturewith PRP | PRP activated by thrombin had ↑HGF, IL-4 and TNF-α  PRP reduced activity of NF-κβ and decreased expression of COX-2 and CXCR4 genes through elevated HGF and TNF-α concentrations  TGF-β1 counteracted TNF-α1 preventing monocyte activation | (+) |
| **Spreafico et al. 2009** [101] | Controlled*in vitro* | Human osteoarthirtic chondrocytes | Chondrocytes cultured with FCS, HS | Histological and immunofluorescence analysis and proteomic study | 2 centrif. /PRP: [Pl]↑ 6,23X and MPC = 1460 ± 292/10^3^ μl/ Thrombin/ TGF-β1 = 126,2 ± 25,2 ng/ml | Chondrocytes cultured wih PRP or PPP at 1%, 5%, 10% | PPR increased chondrocyte proliferation in a dose dependent manner.  PRP increased SOX-9 and aggrecan gene proliferaton.  COL 2 and proteoglican deposition ↑ in PRP treated cultures. | (+) |
| **de Mos et al. 2008** [102] | Controlled*in vitro* | Human tenocytes | Tenocytes cultured in 2% FCS with 0% of PRCR or PPCR | Cell proliferation, total collagen, gene expression (COL1, COL3, MMP1, MMP13, MMP3, VEGF-A, TGFβ1) | 3 centrif. / [Pl] ↑2,55x, [Le] ↓ and [RBC]↓/ CaCl₂/ VEGF, PDGFBB | Culture media suplemented with 10% or 20% PRCR or PPCR | Both releasates (PRCR and PPCR) stimulate cell proliferation and total collagen production. PRCR increased VEGF-4 all and TGFβ1 expression and upregulated MMP1 and MMP3 expression | (+) |
| **Anitua et al. 2007** [103] | Controlled*in vitro* | Synovial cells from OA patients | Culture in serum free media with and without IL-1βstimulation | HA secretion, MMP-1, MMP-3, MMP-13, TIMP-1, TGF-β1, VEGF, | 1 centrif.(specific for PPP or PRP) / CaCl₂ / OA PC = 494 X 10^6^ / TGF-β1 = 38,38 ng/ml/ PDGF-AB = 16,87 ng/ml | I) Non stimulated cells cultured with 20% PP or 20%PRGF  II) Cells cultured with IL1β + PRGF | HA synthesis↑ in PRGF culture. MMPs, TIMP and GFs synthesis were not affected.  PRGF did not halter IL-1βeffects | (+) |
| **Anitua et al. 2006** [104] | Controlled *in vitro* | Human semitendinosus tendons from ACL reconstruction surgery | Culture with fibrinogen, thrombin and CaCl₂ | Synthesis of TGF-β1, VEGF, HGF, cell proliferation, COL1 | 1 centrif.(specific for PPP or PRP) / [Pl]2x↑/ MPC 542 X 10^3^ μL /CaCl₂ | Tendons cultured in PRP and PPP | PRP treated group showed dose dependent increase in cell proliferation, increase VEGF, HGF and TGF Type 1 collagen production was similar in PP- and PR- fibrin matrices, despite lower TGF-β1 concentrations in PP- matrix |  |
|  | Controlled *in vivo* | Sheep (n = 6) | Achilles tendons injected with saline (2.0 ml) weekly for 4 weeks (n = 4) | Histologic evaluation | 1 centrif. or 2 centrif. (PRP or PPP) / CaCl₂ | Injection of preclotted PRP (2.0 ml) in Achilles tendons (n = 4)  Injection of preclotted PPP (2.0 ml) in Achilles tendons (n = 4) weekly for 4 weeks | PP- and PR- matrices increased cell density equally, but signif., compared to control and neovascularization.  Better fiberorganization in PRP treatedtendons. | (±) |
| **Anitua et al. 2005** [105] | Controlled *in vitro* | Human semitendinosus tendons from ACL reconstruction surgery | Culture media alone | Cell proliferation; GFs in culture media | 1 centrif.(specific for PPP or PRP) MPC = 366–642 x 10^6^/ml / CaCl₂ / IGF-1, TGFβ-1, PDGF-AB, VEGF, HGF, EGF | Culture media supplemented with PPP, PPCR, PRCR | Releasates induce synthesis of signif. amounts of VEGF and HGF. PRCR and PPCR induced proliferation of tendon cells | (+) |
| **de Mos et al. 2009** [106] | Descriptive *in vivo* and Controlled *in vitro* | I) Achilles tendinotic lesions and healthy Achilles tendons II) Healthy harmstring and Achilles tendons | I) Healthy Achilles tendons (n = 5)  II) Tendon culture without chondrogenic stimulus | Histologic examination (HE) and RT-PCR (SOX_9_, COL 2A1, aggrecan, COL 10A1, RUNX2) | 2centrif./ [Pl] 2,8x↑/ CaCl₂ | Tendon explants cultured in chondrogenic medial (TGFβ_2_) for 14 days and then cultured with PRP or TA for 7 days. | Chondrogenic differentiation is present in Achilles midportion tendinopathy PRP treated group had decreased expression of chondrogenic differentiation markers | (+) |
| **Xie et al. 2013** [107] | Controlled*in vivo* 12 weeks | Dogs (n = 36; 72 Knees)  ACL reconstruction | I) Saline (n = 18)  II) Shamoperated(n = 18)  III) No surgery (n = 18) | TGFβ1, COL1A1, COL3A1, Decorin, Biglycan, MMP-1, MMP-13 and TIMP-1 mRNA levels by RTPCR | Landesberg’s method / 2centrif./ MPC = 669 ± 313 X 10^9^/L, [Pl] = ↑5X/ CaCl₂ | 1.0 ml PRP (n = 18) | PRP ↑ COL1A1, byglican and MMP-1 expression at all time points compared to saline; PRP ↑COL3A1, MMP-1 and MMP-13 mRNA at 2 and 6 weeks;and ↑TGFβ1 mRNA at 6 and 12 weeks | (+) |
| **Visser et al. 2010** [108] | Controlled*in vitro* | Dogs (n = 4)  Patellar tendons | Cells cultured with blood clot medium | Cell proliferation and viability. Histologic evaluation (HE and Imunostaning for TGF- β1 and GAG) | Cascade Medical Enterprises^11^/ 2 centrif/ CaCl_2_ | Cells cultured with:  I) PRF matrix  II) PRF membrane | PRFibrin groups had ↑ TGF-β1 concentrations (4,7X), ↑cell density, total collagen and GAG. | (+) |
| **Murray et al. 2007** [109] | Controlled *in vivo* | Mongrel dogs (n = 17)  Surgically created wounds in knee intra and extra articular ligaments | I) EA X IA ligament wounds (n = 17)  II) ACL left untreated (n = 5) | Histologicevaluation, immunohistochemistry | 1centrif. | Collagen- PRP hidrogel treated ACL (n = 5) | PRP group had increased GFs and proteins in repair tissue (fibronectin, fibrinogen, PDGF-A, TGF-β1, FGF-2, Von Willebrand factor) and increased wound filling. Histologic differences between EA and IA ligament healing are ameliorated by PRP. | (+) |
| **Fernández-Sarmiento et al. 2013** [110] | Controlled *in vivo* 8 weeks | Sheep (n = 28)  Surgically disrupted Achilles tendons | Saline (n = 14) | Histologic evaluation at 4 and 8 weeks | PRGF -Endoret system^2^/ CaCl₂ | PRGF 4 inj, weekly, 2 ml, US guided (n = 14) | Improvement in morphometric features of fibroblast nuclei in PRGF treated tendons. At 8 weeks PRGF group had more mature organization of collagen bundles, lower vascular densities and ↓ fibloblast densities | (+) |
| **Milano et al. 2012** [111] | Controlled *in vivo* | Sheep (n = 30)  Osteochondral deffects in knee | No treatment after MFx (n = 15) | Macroscopic, histologic (HE and Safranin O) and biomechanical (stiffness) evaluations at 3, 6 and 12 months | ARTHREX®^5^, 1 centrif. / MPC = 868 ± 112 x 10^3^/μl /[Pl] ↑ 2X | 5 weekly inj. of ACP after MFx (n = 15) | PRP group better results in all parameters at all time points, but did not result in hyaline cartilage formation | (±) |
| **Milano et al. 2010** [112] | Controlled*in vivo* | Sheep (n = 15)  Surgically created OC defect in knee | MFx only 12 months after (n = 5) | Macroscopic, biomechanical and histological evaluation (HE/Safranin-O) | 2 centrif. / MPC = 1.415 ± 164 x 10^3^/ml, [Pl] ↑ 4 X / Ca Gluconate + fibrin glue | I) MFx treated with PRP + Fibrin (n = 5)  II) MFx treated with liquid PRP (n = 5)  Treatments performed 12 months after defect creation | Complete healing in MFx + PRPgel group with better macroscopic and histologic features  Almost complete healing in MFx + PRP group  No biomechanical difference between groups  No treatment produced hyaline cartilage | (+) |
| **Yoshioka et al. 2013** [113] | Controlled*in vivo* 6 weeks | Rabbit (n = 31)Medial collateral ligament tear | Non treated tear (n = 14) | Visual, histological (HE, Massom’s trichome), and immunohistochemical examination; biomechanical evaluation, ultimate load and stiffnes at 3 and 6 weeks | PRGF System II^2^ / 1 centrif. /[Pl]↑1,8x, MPC = 39,5 ± 2,4x 10^4^/ μl, WBC free / CaCl₂ / PDGF-BB 0,43 ± 0,14 ng/ml, TGF-β1 2,76 ± 1,83 ng/ml | PRP treatedtears (n = 17) | Improved histologic features and ultimate load in PRGF treated ligaments, with better structural properties at 6 weeks. No signif. difference in in stiffness between groups at 6 weeks. | (±) |
| **Harris et al. 2012** [114] | Controlled *in vivo* | Rabbits (n = 18) | Saline injection | Histology to assess inflammatory cell infiltrate, neovascularization, collagen formation, calcium deposition at 2, 6 and 12 weeks | Platelet Concentrate Collection System^7^ (PCCS)/ CaCl_2_ and thrombin/ 2 centrif., MPC = 1,348.667 ± 427.278/μl | 0.5 ml PRP gel at 0 and 6 weeks in quadriceps muscle, Achilles tendon, medial collateral lig, SC tissue, tibial periosteum and ankle joint | PRP group had collagen bundles present with monocytic and lymphocytic infiltration, thrombosis, necrosis and calcium deposition at injection sites  PRP can initiate an inflammatory response in the abscence in injury  SC sites showed calcium deposition without necrosis and collagen nodules | (−) |
| **Park et al. 2012** [115] | Controlled *in vitro* | Rabbit chondrocytes (n = 6) | Chondrocytes cultured with FBS | Cell proliferation, GAPDH, Aggrecan, COL II, BMP −2 and 7, TGF-β, PDGF, PDGFR-β, VEGF, ChM-I | 2centrif./ MPC = 6 x 10^6^/μl | PRP in culture media at 0.1 a 20% | All PRP concentrations ↑cell proliferation; PRP ↑cellular viability except at 0,1%  SOX-9, TGF-β, VEGF, ChM-1 ↑ with 10% PRP by time dependent manner  PRP ↑stainning for COL II; PRP ↑aggrecan, except at 20%  BMPs and TGF-β ↑ with PRP treatment at 5 and 10% | (+) |
| **Sato et al. 2012** [116] | Controlled *in vivo* | Rabbits (n = 73, 156 tendons)  Intrasynovial flexor tendon tear | No treatment (n = 38) | Edema and adhesion scores at 2, 3, 6 weeks, load to failure, histology | 2centrif / MCP = 313,5 ± 72,3 x 10^4^ / μl | I) 0.5 ml PRP (n = 25)  II) 1.0 μl Fibrin (n = 23)  III) 0.5 μl PRP + 0.5 μl fibrinmatrix (n = 50) | PRP with fibrin matrix ↑ load to failure and had higher histological scores compared to control at 2 weeks  Median histological scores were higher in PRP fibrin matrix group  At 3 and 6 weeks there were no signif. differences beteween groups in load to failure and histological scores | (±) |
| **Lee et al. 2012** [117] | Controlled *in vivo* 8 weeks | Rabbits (n = 20)  ACL reconstruction | Small Intestinal submucosa (SIS) only (n = 10) | ROM, MRI, Biomechanical and histological evaluation | 2 centrif./ Ca gluconate + Thrombin / TGF-β 1 (12 h, 1, 3, 5, 7 days) | SIS group + PRP (n = 10) | PRP group had lower tension load, greater cellular response around grafts and altered cytokine and GFs concentration, which might explain the low tensile strength | (−) |
| **Lyras et al. 2011** [118] | Controlled *in vivo* 4 weeks | Rabbits (n = 48)  Surgically created ACL tears | 0.5 ml saline injection (n = 24) | Histological (HE), immunohistochemical (IGF) evaluation at 1, 2, 3 and 4 weeks | PRP Fast protocol^9^/ 1 centrif. | 0.5 ml PRP (n = 24) | PRP group with longitudinal tenocyte organization and more elongated shape at week 3, with complete healing at week 4.  ↑expression of IGF-1 in PRP group, superior in the epitenon at week 4. | (+) |
| **Lyras et al. 2010** [119] | Controlled *in vivo* 4 weeks | Rabbits (n = 48)  Surgically created patellar tendon defect | Defect left untreated (n = 24) | Histological l (He) and immunohistochemical (spatial and temporal IGF-1 expression) evaluation at 1,2,3 and 4 weeks | PRPFast® protocol^12^/ procoagulant solution/ 1 centrif. | 1.0 ml PRP injectedintralesional (n = 24) | PRP group ↑ intracellular IGF-1 expression at weeks 1 and 2 and after 2nd. week PRP ↑ IGF-1 in tenocytes.  PRP ↑IGF-1 expression in epitenon and endotenon compared with controls.  Fasterhealingobservedin PRP group | (+) |
| **Lyras et al. 2010** [120] | Controlled *in vivo* 4 weeks | Rabbits (n = 48)  Surgically created patellar tendon defect | Defect left untreated (n = 24) | Histological (HE) and immunohistochemical evaluation (anti -CD31) | PRPFast® protocol^12^/ procoagulant solution/ 1 centrif. | 1.0 ml PRP injectedintralesional (n = 24) | PRP group ↑neovascularization only at weeks 1 and 2  PRP stimulated better histological organization, more so at week 3.  At week 4 tendons were completely healed, with no CD31 expression. | (+) |
| **Lyras et al. 2010** [121] | Controlled *in vivo* 4 weeks | Rabbits (n = 48)  Surgically created Achilles tendon defect | Defectleftuntreated (n = 24) | Histological and immunohistochemical evaluation (anti-TGF-β) | PRPFast® protocol^12^/ procoagulant solution/ 1 centrif. | 1.0 ml PRP injectedintralesional (n = 24) | PRP ↑ TGF-β1 gene expression in the first 2 weeks, declining after in comparison with controls | (+) |
| **Saito et al. 2009** [122] | Controlled *in vitro* (I) and *in vivo* (II) | Rabbit chondrocytes (I) and knees with transected ACLs (II) | I) Culture with FBS  II) PBS IA injection | Cartilage mature gene expression, GAG synthesis, histologic and morphological examinations | 2 centrif./ CaCl_2_ and thrombin  MPC in PPP = 6,0 ± 0,5x10^4^/ml MPC in PRP = 1081 ± 149,9 x 10^4^/ml (↑39,4 X) | IA) Culture with 3% PRP  IB) Culture with 3% PPP  IIA) Inj of PBS containing microspheres  IIB) Inj of PRP  IIC) Inj of PRP containing microspheres 4 and 7 weeks postop. | Signif↑ GAG production from chondrocytes cultured in PRP. Signif. ↑expresion of proteoglycan core protein mRNA in articular cartilage. Morphologic and histoloic suppression of OA progression in PRP treated knees (PRP alone and PRP containig microspheres). | (+) |
| **Qi et al. 2009** [123] | Controlled *in vivo* | Rabbits (n = 38)  Cartilage defects in knee joints | Sham operated knees for biomechanical evaluation (n = 5)  Untreated knees (n = 11) | Histologic (HE, Safranin-O), mechanical and morphologic evaluation, ICRS scale at 6 and 12 weeks postop. | 2centrif. / MPC = 18,25 ± 1,21 x 10^5^/μl [Pl]↑7X | Deffects treated with:  I) Bylayer collagen matrix (n = 11)  II) Bylayer collagen matrix + PRP (n = 11) | Signif. improvement in ICRS histological scores and ↑ GAG content in PRP treated matrix.  No difference in biomechanical tests between treated groups groups and results from both were inferior to control’s. | (±) |
| **Lyras et al. 2009** [124] | Controlled *in vivo* | NZ white rabbits (n = 48)  Surgically transected Achilles tendons | Saline injection (n = 24) | Histologic evaluation (HE) and immunohistochemistry (CD 31 marker) | PRP Fast system^12^/ 1 centrif. | PRP inj. (n = 24) | PRP treated group had signif. ↑ Vascularization at weeks 1 and 2 which decreased at week 4, also signif. At week3 PRP grouphadbetterhistologicalorganization. | (+) |
| **Lyras et al. 2009** [125] | Controlled *in vivo* | NZ white rabbits (n = 52)  Surgically transected Patellar tendons | Defect left untreated (n = 26) | Histologic (HE) and mechanical evaluation (load to failure, stiffness, energy uptake) | 1 centrif. / MPC = 4248 ± 1132 X10^3^/μl / [Pl]↑ 8x / PDGF-BB = 22,32 ± 9,2 ng/ml TGF-β1 = 199,37 ± 36,62 ng/ml, VEGF = 957,46 ± 328,52 ng/ml, EGF = 462,87 ± 271,69 ng/ml / Precoagulation solution | Deffect filled with PRP (n = 26) | At 2 weeks PRP treated tendon had signif. improved mechanical properties.  At 4 weeks there were no differences between groups. At 28 days PRP treated tendons had ↑collagen deposition and were completely healed, while control tendons were not | (+) |
| **Sun et al. 2009** [126] | Controlled *in vivo*  12 weeks | NZ White rabbits (n = 24)  Surgically created full- thickness OC defect on patelar groove | Defects left untreated (n = 8) | Macroscopic and histological assessment, micro-CT image | 2 centrif./ CaCl_2_ and thrombin / MPC = 125.59 10^4^ μL/ [Pl]↑512 X / GFs analysis | PRP filled defects (n = 16) | Macroscopic improvement.  Better histological apperance and histologycal scoring in PRP trated group.  At 12 weeks PRP group had larger amount of subchondral bone formation | (+) |
| **Wu et al. 2007** [127] | Controlled *in vivo* | NZ whiterabbits (n = 8)  Auricular chondrocytes | Only PRP injected SC (n = 4) | Hystologic evaluation, macroscopic examination, GAG content and MRI | 2centrif./ Thrombin, CaCl₂ | PRP + chondrocyte injected SC (n = 4) | Cartilaginous tissue present in SC masses in MRI and histologic evaluation (presence of GAG and collagen)in PRP group only. | (+) |
| **Dallaudière et al. 2013** [128] | Controlled*in vivo* 25 days | Rats (n = 30)  120 colagenase-induced tendinosis in patellar and Achilles tendons | I) Saline (efficacy) (n = 40)  II) Not injected (toxicity) (n = 40) | Clinical, US and histologic evaluation | 1 centrif./ MPC =1.500.000 ± 42.000 in final volume, [Pl] ↑3X/ ↓ Le | I) 40 intralesional inj. 0.1 ml PRP, US guided (efficacy)  II) 40 healthy ligs. Inj. with PRP (toxicity) | PRP group had better mobilization, ultrasonographic and histologic features at all time points, compared to saline. No observedtoxicity. | (+) |
| **Kaux et al. 2012** [129] | Controlled*in vivo* | Rats (n = 132)  Sectioned Achilles tendons | 50 μl Saline injected (n = 60) | Biomechanical, histological (HE and Masson’s trichrome) and biochemical (tenomodulin, COL1, COL3, MMP −2,MMP-3 and MMP-9 expression) evaluation | Plateltex®^3^, 2 centrif/ MPC = 2,2 a 2,9 X 10^6^ / mm^3^ / [Pl] 3-4X ↑, very few WBC or RBC / CaCl₂ | 50 μl PRP intralesionalinjection | ↑COL 1 expression at day 30 and tenomodulin at day 5, in PRP group  Tendons in PRP group had higher ultimate tensile strenght at all time points and were more resistant to mechanical stress at day 30  Larger tendon area PRPgroup from day 5 to 15, normal at day 30  PRP group showed precocious deposition of fibrilar collagen | (+) |
| **Vaisman et al. 2012** [130] | Controlled*in vivo* | Rats (n = 13)  Osteochondral defects in 52 femoral condyles | Unlesioned, untreated group (n = 12) | Macroscopic and hystologic (HE) evaluation; molecular analysis (COL II and COL I gene expression) | 2 centrif/ MPC = 1,5X10^6^/μl / CaCL₂ / Flow cytometry analysis | I) Untreated lesions (n = 8)  II) MFx treated lesions (n = 8)  III) MFx + BMS treated lesions (n = 12)  IV) MFx + PRP treated lesions (n = 12) | BMS and PRP as coadjuvants to MFx technique are not associated with improvement in hyaline cartilage regeneration, although macroscopic improvement was observed | (±) |
| **Hapa et al. 2012** [131] | Controlled *in vivo* 4 weeks | Rats (n = 83)  Rotattor cuff tear | Saline treated lesion 2 weeks (n = 17)  Saline treated lesion 4 weeks (n = 17) | Histological and biomechanical evaluation at 2 and 4 weeks | 2 centrif/ MPC 13.8 X 10^9^/L | I) PRP treated group 2 weeks (n = 17)  II) PRP treated group 4 weeks (n = 17) | Less inflammatory cells and angiogenesis in PRP treated lesions.  Better tendon thickness and continuity at 2 weeks than controls  Treated tendons did failure a at higher load than controls at 2 weeks | (+) |
| **Beck et al. 2012** [132] | Controlled *in vivo* 21 days | Rats (n = 102)  Rotator cuff repair | Repair only (n = 15)  Sham-surgery (n = 6) | Biomechanical testing (failure load, stiffness, strain)  Histological analysis | 2 centrif/ [Pl] ↑ 3–5 X/ Cacl_2_ and thrombin | I)Repair with PRP (n = 15) II) Repair with PRP at 7d (n = 15)  III)Repair with PRP 14 d (n = 15)  IV)Repair with PRP 21 d (n = 15)  [Pl]/treatment = 4,4 X blood | Three rats with postoperative infections,1 in 14d group and 2 in the 21d group  At 7 days PRP group had↓ tissue strain and cellular infiltration, lack of collagen orientation and fibrinoid necrosis  At day 14 no mechanical differences between treated and control groups and PRP group had hypertrophic chondrocytes  At day 21 PRP group had ↑ failure strain | (−) |
| **Spang et al. 2011** [133] | Controlled *in vivo* 14 days | Rats  Surgically created Patellar tendon lesions | 0.5 ml saline injection (n = 22) | Histological (HE) and biomechanical testing (load -deformation curve, stifness, energy absorved and ultimate tensile load) | ARTHEX®5/ 1 centrif./ variable platelet counts after processing (↑ ≤ 2x) | 0.5 ml PRP on lesions (n = 25) | No differences between groups  Highest platelet counts in PRP related to higher ultimate tensile load and energy absorbed to failure | (±) |
| **Tohidnezhad et al. 2011** [134] | Controlled *in vitro* | Tenocytes isolated from Achilles tendon of postnatal rats | Tenocytes in control medium | WST assay (growth and viability), scratch test (migration + proliferation), luciferase assay, immunohistochemistry | 2centrif/ [Pl] 5-10x↑/ CaCl₂ | Addition of 10% PRGF to culture medium | PRP had positive effect on tendon growth, proliferation, viability and migratory capacity. PRP activatedanantioxidant response | (+) |
| **Kajikawa et al. 2008** [135] | Controlled *in vivo* | Transgenic Rats GFP chimeric and Sprague Dawley  Surgically created patellar tendon lesions | Lesionsnotinjected | GFP-positive cells and morphometric analysis, histologic evaluation (HE), immunohistochemity (COL1, COL3), macrophage and cell proliferation | 2 centrif. / [Pl] ↑ 8,8x/ MPC = 523,8X 10^4^/μl TGFβ1 = 62,3 X 10^2^ ng/ml, PDGF-BB = 22,2 ng/ml | Lesionsinj. with PRP | PRP treated tendons had increased collagen production and greater rate of macrophage proliferation and participation of circulation-derived macrophages in the early phase of tendon remodeling | (+) |
| **Virchenko; Aspenberg 2006** [136] | Controlled*in vivo* | Sprague–Dawley Rats (n = 130) Achilles tendon transection model | I) Botox + saline  II) Ordinary cages + no Platelet gel  III) Activity cages + no Platelet gel | Mechanical evaluation (load to failure, stiffness energy uptake, stress at failure) and transverse area at 3, 5 and 14 days. | 2 centrif./ MPC 8,3x10^9^/l / Thrombin and CaCl₂ | Platelet gel +  I) Botox  II) Normal cages  III) Activity cages | Platelet gel promoted tendon repair while unloading abolished its stimulatory effect, and reduced mechanical properties of repair tissue. | (+) |
| **Aspenberg; Virchenko 2004** [137] | Controlled*in vivo* | Sprague–Dawley Rats (n = 296)  Achilles tendon transection model | Buffer injection | Mechanical evaluation (force at failure, stiffness, energy uptake) and histological evaluation at 8, 11, 14, 21 and 28 days | 2 centrif. / MPC = 1,5x10^12^/L / Thrombin | PC injection | PC injection ↑tendon callus strength and stiffness, effect that persisted 3 weeks after treatment. Improvement in histological scores and material characteristics of tendon callus, with signif. more mature callus at 21 days in PC treated tendons | (+) |
| **Murray et al. 2009** [138] | Controlled *in vivo* | Immature Yorkshire pigs (n = 6)  Bilaterally transected ACLs | ACL suture repair (n = 6) | Anterior - posterior knee laxity, tensile load properties and stiffness | 2centrif./ [Pl]↑ 2,83 ± 0,53x; [Le] ↑ 1,95 ± 0,34 | ACL suture repair + 3.0 ml of PRP (n = 6) | No improvement in any of the outcome measures at 14 weeks | (−) |
| **Murray et al. 2007** [139] | Controlled*in vivo* | Yorkshire pigs (n = 5)  Surgically transected ACL | Suture repair only (n = 11) + intact knees (n = 6) | MRI, biomechanical tests and histologic evaluation | 1centrif. / MPC =954.000 ± 93.000 / mm^3^ | Suture repair with collagen PRP hydrogel (n = 5) | Signif. improvements in biomechanical properties in PRP treated ligaments at 4 weeks. Structural properties of PRP treated ligaments were positively affected, but remained signif. inferior to the intact group.  Highly cellular repair tissue within defect at 4 weeks | (+) |
| **Akeda et al. 2006** [140] | Controlled *in vitro* | Minipigs chondrocytes (n = 8) | Condrocytescultured in FBS | Cell proliferation (DNA content), proteoglycan synthesis and total content, collagen synthesis | Symphony 2 Platelet Concentration System^13^ (PRP and PPP) / MPC = 1393 ± 174 x 10^3^/ml / Thrombin/ TGF-β1 in serum and releasates | Condrocytes cultured in:  FBS 10%  PPP 10%  PRP 10% | PRP increased condrocyte cellular proliferation, proteoglycan and COL 2 synthesis, and total proteoglycan content. | (+) |

[]:concentration; ↑: increase;↓:decrease;

^1^Pall, Port Washington, New York, USA; ^2^ Endoret BTI Biotechnology, San Antonio, Spain; ^3^Plateltex S.R.O, Vlasska, Praga, CZ; ^4^ Harvest Technologies Corporation, MA, 02360, USA; ^5^ Arthex Naples, Florida, USA; ^6^ Terumo BCT, Mississauga, Ontario, USA; ^7^ Biomet Corporate Headquarters, Palm Beach Gardens, Florida, USA; ^8^Biomet Corporate Headquarters, Warsaw, Indiana, USA; ^9^Biomet Corporate Headquarters, Warsaw, Indiana, USA; ^10^ Haemonetics Corporation, Braintree, MA, USA; ^11^ MTF Sports Medicine Edison, New Jersey, USA; ^12^ Bioteck, Vicenza, Italy; ^13^ DePuy Spine, Raynham, MA, USA.
